# Dual species transcriptomics reveals conserved metabolic and immunologic processes in interactions between human neutrophils and *Neisseria gonorrhoeae*

**DOI:** 10.1371/journal.ppat.1012369

**Published:** 2024-07-08

**Authors:** Aimee D. Potter, Vonetta L. Edwards, Adonis D’Mello, Mary C. Gray, Amol C. Shetty, Amy L. Forehand, Camille S. Westlake, Evan R. Lamb, Xuechu Zhao, Stephanie A. Ragland, Alison K. Criss, Hervé Tettelin

**Affiliations:** 1 Department of Microbiology, Immunology, and Cancer Biology, University of Virginia School of Medicine, Charlottesville, Virginia, United States of America; 2 Institute for Genome Sciences, Department of Microbiology and Immunology, University of Maryland School of Medicine, Baltimore, Maryland, United States of America; University of Basel, SWITZERLAND

## Abstract

*Neisseria gonorrhoeae* (the gonococcus, Gc) causes the sexually transmitted infection gonorrhea. Gc is a prominent threat to human health by causing severe lifelong sequelae, including infertility and chronic pelvic pain, which is amplified by the emergence of “superbug” strains resistant to all current antibiotics. Gc is highly adapted to colonize human mucosal surfaces, where it survives despite initiating a robust inflammatory response and influx of polymorphonuclear leukocytes (PMNs, neutrophils) that typically clear bacteria. Here, dual-species RNA-sequencing was used to define Gc and PMN transcriptional profiles alone and after infection. Core host and bacterial responses were assessed for two strains of Gc and three human donors’ PMNs. Comparative analysis of Gc transcripts revealed overlap between Gc responses to PMNs, iron, and hydrogen peroxide; 98 transcripts were differentially expressed across both Gc strains in response to PMN co-culture, including iron-responsive and oxidative stress response genes. We experimentally determined that the iron-dependent TbpB is suppressed by PMN co-culture, and iron-limited Gc have a survival advantage when cultured with PMNs. Analysis of PMN transcripts modulated by Gc infection revealed differential expression of genes driving cell adhesion, migration, inflammatory responses, and inflammation resolution pathways. Production of pro-inflammatory cytokines, including IL1B and IL8, the adhesion factor ICAM1, and prostaglandin PGE2 were induced in PMNs in response to Gc. Together, this study represents a comprehensive and experimentally validated dual-species transcriptomic analysis of two isolates of Gc and primary human PMNs that gives insight into how this bacterium survives innate immune onslaught to cause disease.

## Author summary

The bacterium *Neisseria gonorrhoeae* (Gc) is a public health threat with emerging resistance to antibiotics. Gc induces robust mucosal inflammatory responses with abundant influx of white blood cells called polymorphonuclear leukocytes (PMNs, neutrophils). While PMNs are highly antimicrobial, Gc survives amongst PMNs for reasons incompletely understood. Here we detail the transcriptional profiles of PMNs from multiple human donors and Gc strains, to investigate this host-pathogen interaction in a comprehensive, unbiased manner. The core transcriptome across Gc exposed to PMNs overlaps transcriptomes of Gc exposed to stresses imparted by immune pressure, specifically nutrient metal starvation. Additionally, core host pathways induced upon PMN exposure to Gc include surface and secreted factors that enable PMN migration and activation. This study uncovers critical features of the Gc-PMN interface to inform how Gc resists innate immune clearance.

## Introduction

Gonorrhea, caused by the bacterial pathogen *Neisseria gonorrhoeae* (gonococcus, Gc), is one of the most common sexually transmitted infections in the United States and globally [1,2]. Drug resistant gonorrhea is an emergent threat to global health, as Gc has rapidly acquired resistance to antibiotics including β-lactams, tetracyclines, and fluoroquinolones [3–5]. Intramuscular administration of ceftriaxone is the only current frontline antibiotic treatment recommended for uncomplicated gonococcal infection in the United States [6], and high-level ceftriaxone-resistant strains of Gc have been isolated throughout the world, highlighting the urgent need for new treatments [7–10]. Approximately half of infections in females are asymptomatic [11], which can result in a high number of untreated infections that may lead to severe clinical sequelae such as pelvic inflammatory disease, ectopic pregnancies, and infertility [12,13].

Many sequelae from gonococcal infection are associated with a sustained and unresolved inflammatory response featuring polymorphonuclear cells (neutrophils, PMNs). PMNs can be recruited by interleukin 8 (IL8), IL6, IL1, and tumor necrosis factor-alpha (TNFα), which are produced during gonococcal infection [14–16]. PMNs are directly involved in modulating the innate immune response within the host through the release of proteases, antimicrobial peptides, and reactive oxygen species (ROS), which are delivered to pathogens from cytotoxic granules during phagocytosis and in neutrophil extracellular traps (NETs) [17]. However, intact Gc are observed within and attached to PMNs in male urethral exudates and female cervical secretions, and Gc can be cultured from these specimens [18]. Gc evades PMN clearance by undergoing antigenic and phase variation to avoid antibody-mediated opsonization, expressing gene products that defend against toxic PMN species, modulating their delivery to phagolysosomes, and delaying the spontaneous apoptosis of PMNs [18–20]. Moreover, while the historical consensus is that the PMN population is homologous with well-defined and conserved functions, recent transcriptomic analysis has shown that there is both heterogeneity and functional versatility within this population [21]. Our field currently lacks a comprehensive understanding of the mechanisms Gc and PMNs direct against each other, and how Gc prevails.

It is highly unusual for a pathogen to survive exposure to PMNs [19]. To systematically investigate the mechanisms PMNs direct against Gc, and conversely that Gc uses to resist PMN clearance, we performed dual-species RNA-seq transcriptional profiling of two strains of Gc interacting with adherent, IL8 primed primary PMNs from three unrelated individuals. Our study of host and pathogen genes, functions, and pathways associated with Gc-PMN interactions provides the first detailed transcriptomic analysis of the response of PMNs from multiple donors to different Gc strains and vice-versa.

## Results

### Modeling Gc-PMN interactions during human infection

We investigated the conserved transcriptional responses of two Gc strains and primary PMNs from three unrelated individuals, alone and in co-culture. To investigate interactions of Gc with human PMNs, we used an infection model with adherent, IL8-treated primary human PMNs to mimic the tissue-migrated state of PMNs in human disease [22,23]. Neutrophils represented over 95% of the purified PMN cell population (**S1 Fig**). We used two Gc isolates: a constitutively piliated, Opa^-^ derivative of strain FA1090 (Opaless clone 130, called hereafter FA1090 Opaless); and H041 (WHO X), a multidrug-resistant clinical isolate [10,24]. FA1090 Opaless and H041 Gc strains exposed to PMNs displayed an initial decrease in viability at 30 min, followed by a recovery and outgrowth period at 1h post infection (**S2A Fig**), compared to a constitutively Opa-expressing FA1090 derivative, which is readily killed by PMNs (**S2A Fig**). We examined the transcriptional responses of Gc and PMNs at the start of the outgrowth period to define the transcriptome of Gc that is resistant to PMN killing and the response of PMNs towards replicating Gc. Samples were harvested for RNA extraction at the time of Gc addition to PMNs (Gc+PMN_0h) and after 1h of infection (Gc+PMN_1h). For comparisons, we included PMNs without infection at 0h (PMN_0h) and 1h (PMN_1h), and Gc incubated without PMNs for 1h (Gc_1h). Gc added to PMNs at 0h did not undergo any incubation and were used to represent the baseline for Gc at 0h. Sufficient RNA sequencing depth for saturated detection of expressed Gc and PMN genes was confirmed using rarefaction curves (**S3 Fig and S1 Dataset**).

### Infection drives consistent differences in gene expression profiles of Gc and human PMN transcriptomes across strain background and PMN donors

To compare gene expression across Gc strains, orthologous protein coding genes were determined among FA1090 (used for annotation purposes), FA1090 Opaless, and H041 genome sequences using the Pan-genome Ortholog Clustering Tool (PanOCT) [25]. Gene ortholog information was then visualized using a Venn diagram (**S4 Fig**), showing core genes shared by all three strains (1636) and the different strain-specific accessory genes or those shared by different pairs of strains. 120 protein coding genes (~6% of H041 protein coding genes) were strain-specific accessory genes for H041 not found in FA1090 or FA1090 Opaless. Conversely, 157 protein coding genes (~7.7% of FA1090 Opaless protein coding genes) were strain specific accessory genes annotated in FA1090 Opaless but not found in H041. From the 1636 PanOCT clusters of orthologs identified, clusters harboring multiple genes from the same genome (e.g., paralogs or gene fragments) were filtered out as they would confound downstream analysis, resulting in 1582 clusters of core genes used for combined transcriptome analyses (**S2 Dataset**).

**Fig 1:**
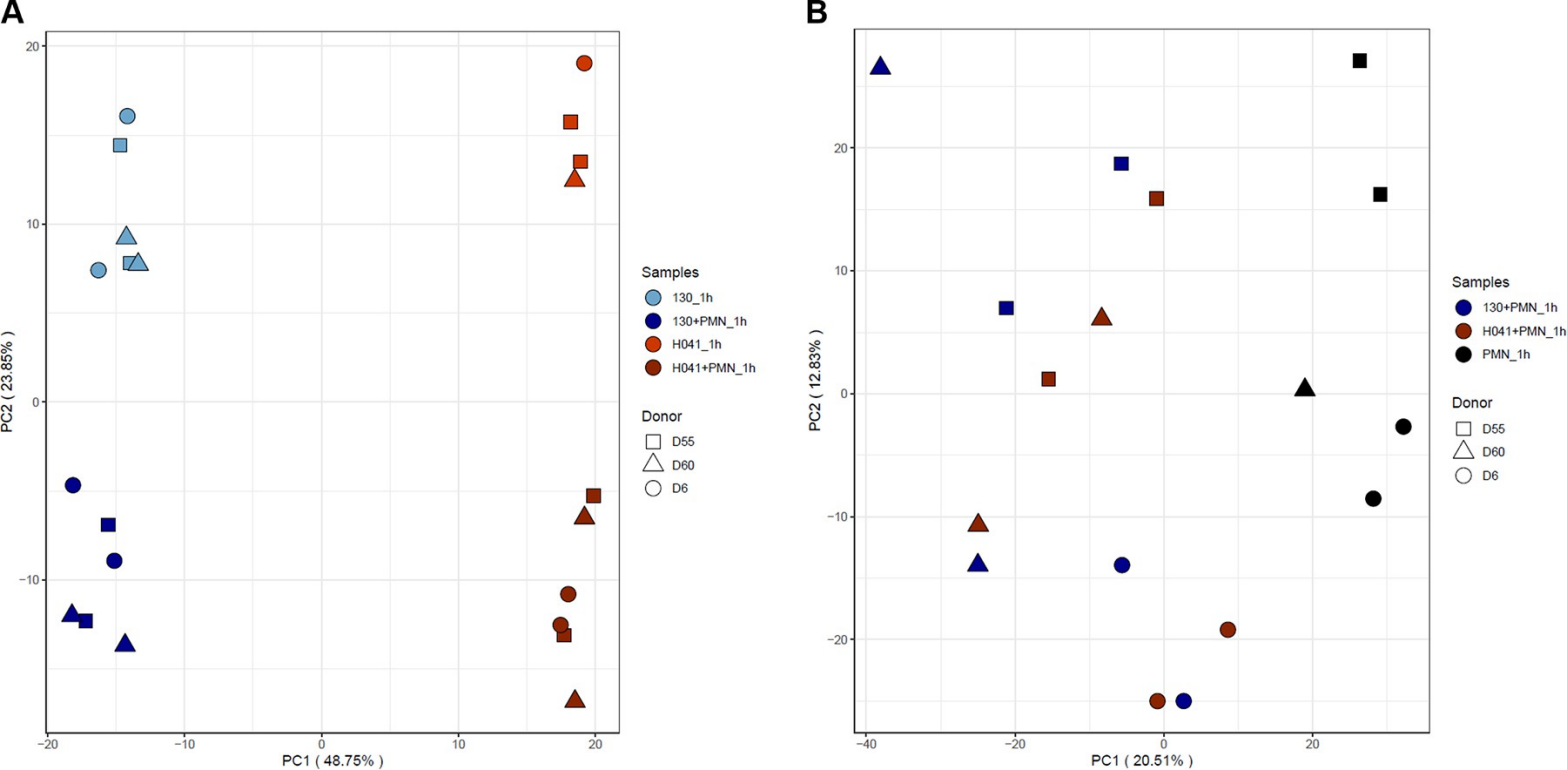
Principal Component Analyses (PCA) of Gc and PMN transcriptomes. A) PCA of Gc transcriptomes based on 1582 core genes. PC1 separates the 130 and H041 strains, and PC2 separates Gc infected PMN samples from Gc in culture alone. B) PCA of PMN transcriptomes. PC1 separates infected PMN samples from uninfected PMNs, and PC2 separates the different PMN donors with D55 trending towards the top, D60 primarily in the middle, and D6 towards the bottom.

We observed distinct expression profiles for Gc core genes and PMN genes by principal component analysis (PCA) (**S2 Dataset, Figs 1 and S5**). Variation in Gc and PMN responses were associated with time of incubation (1h vs 0h) (**S5 Fig**). Therefore, further analyses were performed only at 1h between mono-cultured samples and Gc-PMN co-cultured samples. The majority of the Gc response within core genes was driven by strain background (**Fig 1A** PC1, 48.75% of variation), followed by experimental condition (PC2, 23.85%). Percent variations captured by PCs 3 and above were all <10%, indicating that PCs 1 and 2 captured most of the biologically relevant features of the Gc transcriptional profiles. This stark separation by Gc strain is expected when analysis is performed using core bacterial genes from different backgrounds [26] and highlights the transcriptomic differences between high passage reference strains (e.g. FA1090, the parent of Opaless) and clinical isolates (e.g. H041). Biological replicates within each Gc strain and infection condition clustered together, indicating the Gc response was generally conserved. Similarly, PCA was performed on PMN samples (**Fig 1B**). Variation in PMN response was associated with infection with Gc (PC1, 20.51%) and loosely with donor-specific responses (PC2, 12.83%). Percent variations captured by PCs 3 and above were <10% as well for PMNs. Given that primary human PMNs from unrelated donors were used, donor-to-donor variation was expected. For this reason, we used donors as a covariate in all downstream differential expression testing. To identify the genes that most likely contributed to human PMN variation, we used the union of the top 1% of genes from PCs 2, 3, and 5 (<1500 genes) to produce a 3-dimensional PCA which clusters the 3 different donors (**S5C Fig**). A list of these genes is provided in **S2 Dataset**.

### Differential regulation of selected Gc gene subsets in response to PMNs

**Fig 2:**
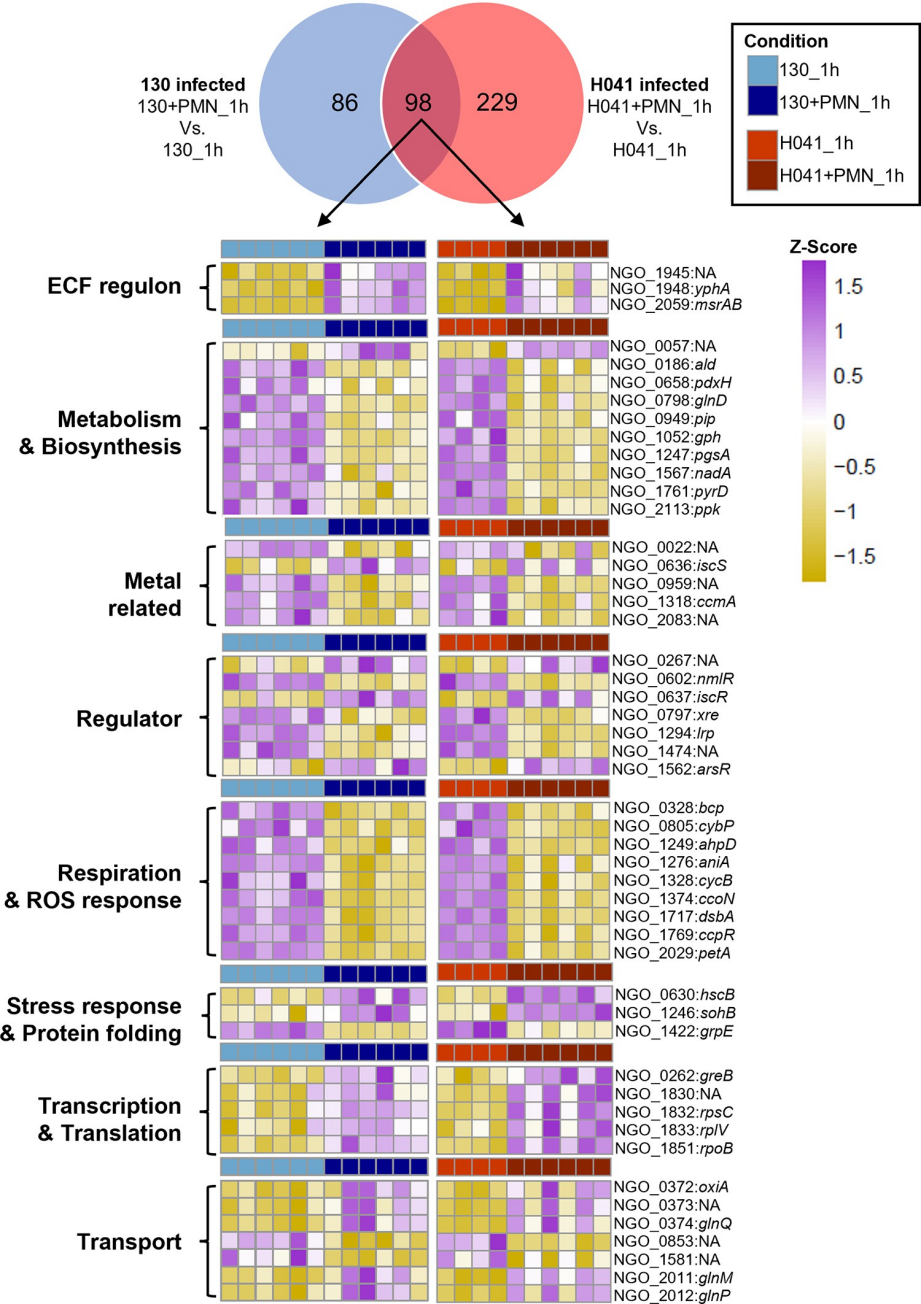
Venn diagram of differentially expressed (DE) Gc genes (Dataset S3) and Z-scored heatmaps of selected Gc regulons showing expression patterns for DE genes shared by strains FA1090 Opaless 130 and H041. Colors indicate Z-score normalized expression values. The 98 shared DE genes (i.e. DE in 130 and H041, grouped using a one-to-one orthology) classified into major Gc regulons and heatmaps are presented for both species. Heatmaps of strain FA1090 Opaless 130 and H041 specific DE genes are shown in **S6** and **S7 Figs**.

**Fig 3:**
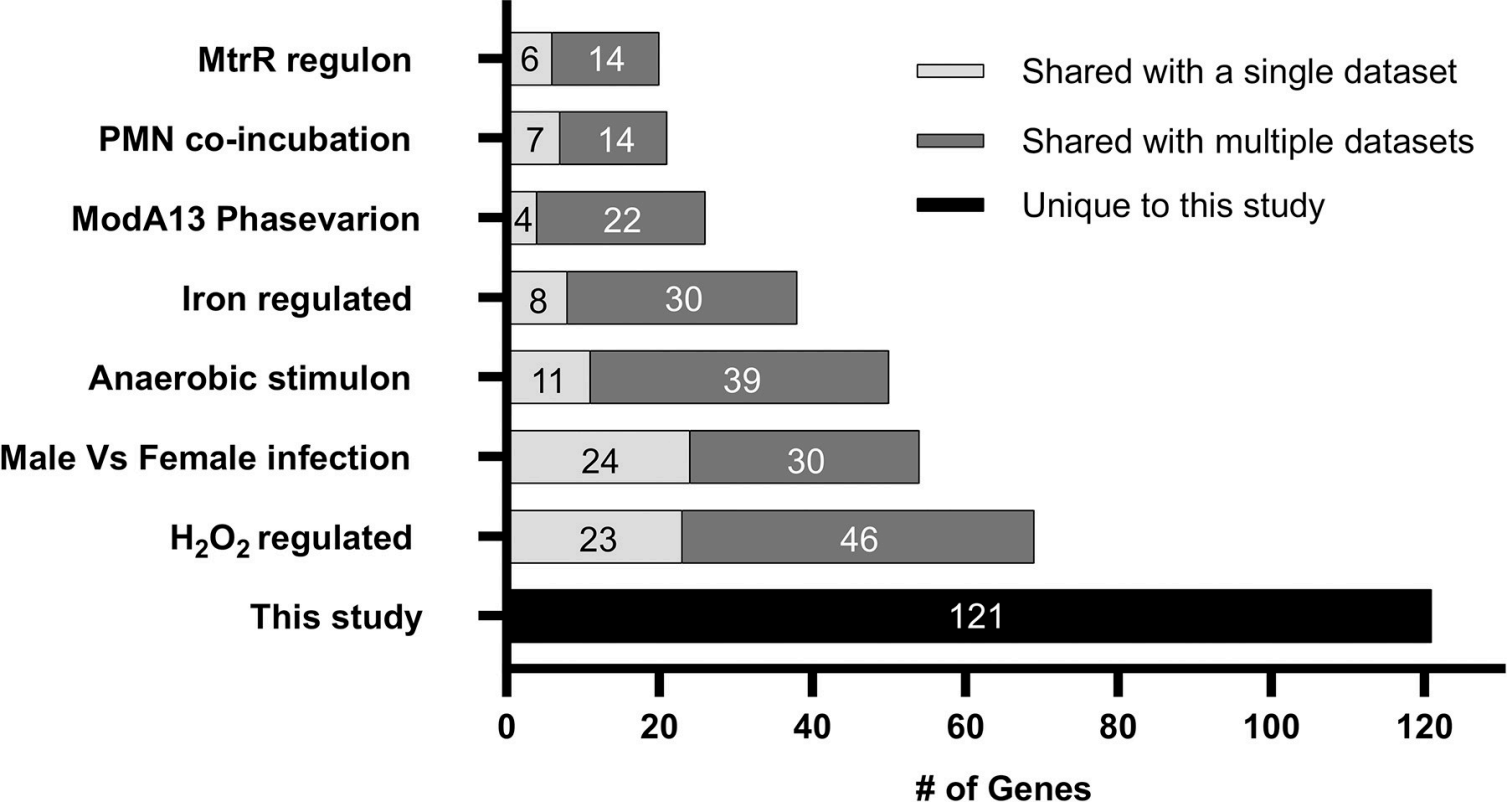
Comparison of Gc differentially expressed (DE) genes identified in this study to other transcriptomics datasets (Dataset S3). Total lengths of bars represent the number of genes identified in this study. Light grey bars represent genes that were only identified in the Gc response to PMNs (this study) and the indicated dataset. Dark grey bars represent genes that were identified in multiple datasets. The black bar represents genes that were not identified in other studies.

Examining the response of FA1090 Opaless and H041 to adherent PMNs (Gc+PMN_1h vs Gc_1h), we identified 86 differentially expressed (DE) genes specific to FA1090 Opaless Gc, 229 DE genes specific to H041 Gc, and 98 genes DE in both strains in response to PMNs (total: 413 genes) (**Fig 2**). All strain-specific responses between FA1090 Opaless and H041 were due to differences in the cutoffs that were set to determine significant differential expression, and none exhibited opposing regulation with the exception of a single gene (predicted transposase; FA1090 Opaless: F9Z35_0257; H041: F9Z36_1237). For the 413 genes, expression patterns were often maintained between Opaless FA1090 and H041, but below the threshold for significance (**S6 and S7 Figs**). Of the 413 DE genes, 290 could be mapped to corresponding NGO IDs from the original FA1090 genome with associated functional categories from literature surveys. Strikingly, 30% of DE genes had roles in metabolism, including central metabolism, amino acid metabolism, cofactor synthesis, and nutrient transport (**S3 Dataset**). The metabolic adaptations Gc undergoes during PMN co-culture were assessed in depth in a corresponding study [27]. The 413 genes were compared to RNA-seq datasets interrogating other infection-related factors including iron, hydrogen peroxide (H_2_O_2_), anaerobic growth, exposure to PMNs in suspension, and natural human infection (**Fig 3 and S3 Dataset**) [28–32]. The Gc response to PMNs overlapped substantially with the anaerobic, H_2_O_2_, and iron responses with 50, 69, and 38 of the 413 DE genes shared, respectively (**Fig 3** and **S3 Dataset**).

### Gc upregulates oxidative stress response genes upon exposure to PMNs

Of the 413 Gc genes DE upon exposure to PMNs, 69 were previously reported as responsive to H_2_O_2_ (**Fig 3** and **S3 Dataset**) [29]. Reads mapping to methionine sulfoxide reductase polyprotein (*msrAB*, NGO2059) exhibited one of the greatest increases in abundance upon exposure to PMNs in both Gc strains (Log_2_ Fold Change FA1090 Opaless: 4.64; H041: 3.08) (**S3 Dataset**). MsrA/B is associated with oxidative damage repair in Gc and is required for survival from oxidative stressors including H_2_O_2_ and superoxide anions [33]. *msrAB* is regulated by the alternative sigma factor Ecf (NGO1944). *ecf*, *msrAB*, and members of the *ecf* operon (NGO1944, NGO1945, NGO1946, and NGO1948) were upregulated in Gc exposed to PMNs in both Gc strains (**Figs 2 and S6**) [34]. We speculated that an Δ*ecf* mutant may have defects in the presence of PMNs, particularly in the context of oxidative stress. As FA1090 Opaless does not induce an oxidative burst from PMNs, we assessed survival of a Gc Δ*ecf* mutant upon exposure to PMNs in both the FA1090 Opaless or an FA1090 derivative constitutively expressing OpaD, which induces an oxidative burst from PMNs (**S2B–S2C Fig**). Survival of an isogenic Δ*ecf* mutant exposed to PMNs was unchanged compared to the FA1090 Opaless or Opa^+^ parent (**S8 Fig**). The contribution of the *ecf* operon on survival of Gc from PMNs thus remains unclear.

In addition to *msrAB* and the *ecf* regulon, other H_2_O_2_-responsive transcripts were also more abundant in PMN-exposed Gc (**Fig 3** and **S3 Dataset**) [29]. The response to H_2_O_2_ is multifactorial and overlaps significantly with other datasets, including the iron regulon and anaerobic regulon in Gc [30,31]. Nutrient homeostasis, particularly nutrient metals, can affect oxidative stress resistance [20]. As such, of the 69 H_2_O_2_-responsive genes that are also more abundant in PMN-exposed Gc, 18 also encode iron-responsive proteins (NGO0208, NGO0226, NGO0322, NGO0554, NGO0632-*iscA*, NGO0633-*iscU*, NGO0635, NGO0636-*iscS*, NGO0637-*iscR*, NGO0754-*mobA*, NGO0863, NGO0929-*metF*, NGO1029-*fumC*, NGO1215, NGO1276-*aniA*, NGO1277, NGO1318-*ccmA*, and NGO1769-*ccpR*). The remaining 51 H_2_O_2_-responsive genes that are also more abundant in PMN-exposed Gc encode nutrient acquisition proteins (NGO0377-*citT* and NGO1205-*tdfJ*), metabolic proteins (NGO1931-*gapC*, NGO0798-*glnD*, NGO1247-*pgsA*, NGO1382-*relA*, NGO1671-*coaE*, and NGO2113-*ppk*), phage associated ORFs (NGO0489-*rusA*, NGO0509, NGO1002-*traA*, NGO1090-*gp56*, NGO1116-*prtR*, and NGO1630), transcriptional regulators (NGO0797-*xre*, NGO1244-*marR*, and NGO1294-*lrp*), respiration and ROS response proteins (NGO1189-*hslO*, NGO1249-*ahpD*, NGO1328-*cycB*, NGO1371-*ccoP*, and NGO1442-*adhA*), stress response and protein folding (NGO0116-*secB*, NGO0630-*hscB*, NGO0829-*hscA*, NGO1046-*clpB*, NGO1422-*grpE*), transcription and translation proteins (NGO0191-*rpsO*, NGO0295-*thrS*, NGO0583-*rpsR*, NGO0899-*greA*, NGO1284-*rimP*, NGO1337-*prfA*, NGO18261-*rpsN*, NGO1832-*rpsC*, NGO1835-*rplB*, NGO1836-*rplW*, NGO1837-*rplD*), restriction-modification proteins (NGO0545), surface protein NGO0223-*nspA*, and ORFs of unknown function (NGO0569,NGO0711, NGO0788, NGO0827, NGO0901, NGO1370, and NGO2162).

Based on these results, we hypothesized that Gc exposure to PMNs primes an oxidative stress response that enhances bacterial survival from ROS. To test this, FA1090 Opaless Gc, which do not induce a PMN oxidative burst (**S2B–S2C Fig**), were exposed to PMNs for 1h or medium alone, then treated with increasing concentrations of H_2_O_2_ for 15 min. Exposure to PMNs modestly (~12.6%) but statistically significantly increased the percent of Gc recovered after H_2_O_2_ treatment, indicating that the transcriptional program initiated by PMN challenge helps defend against oxidative stress (**S9 Fig**).

### Differential expression of metal acquisition systems indicates ways Gc uses to overcome nutritional immunity when exposed to PMNs

**Fig 4:**
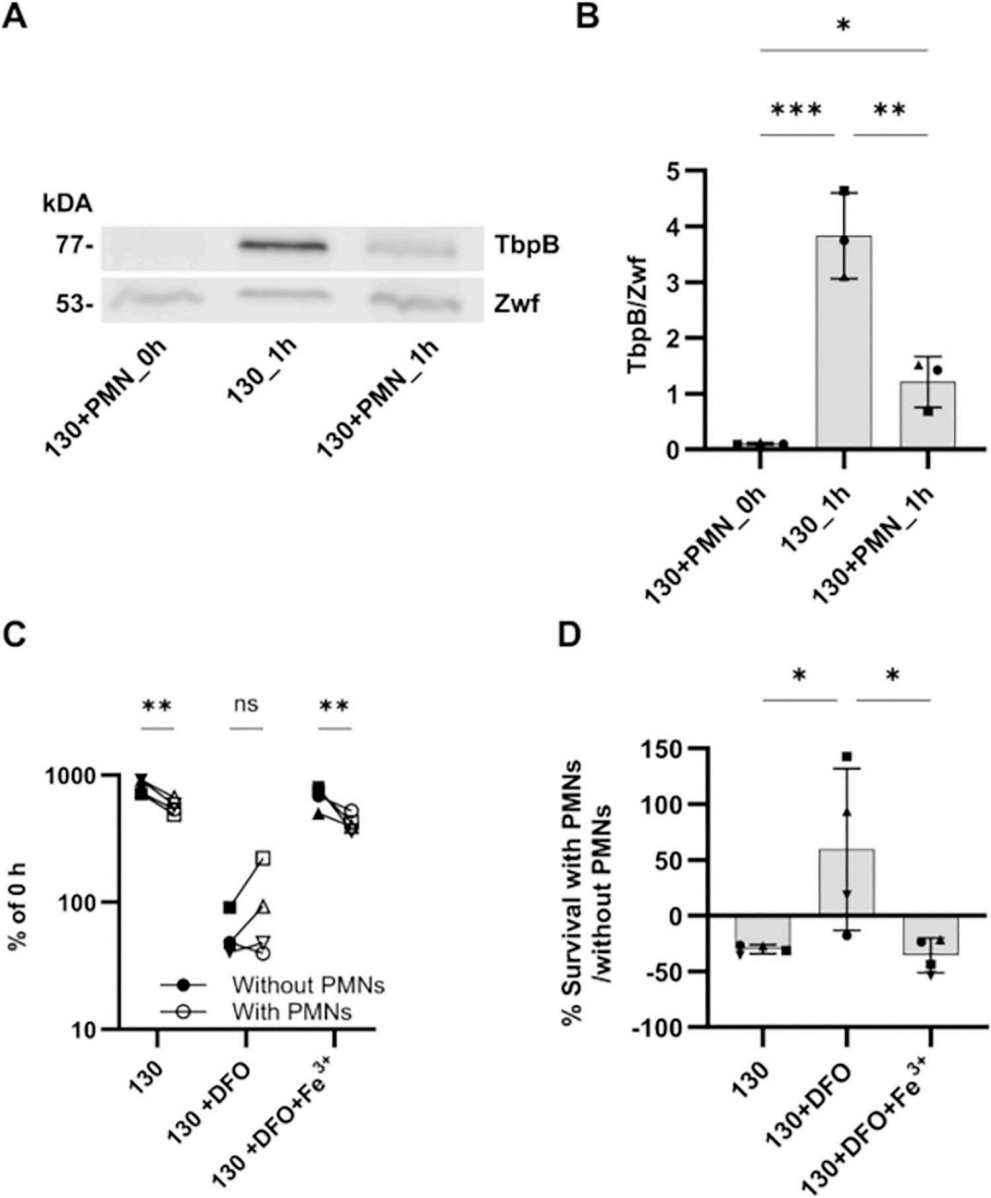
Gc exposed to PMNs exhibit production of an iron-regulated metal acquisition protein and increased survival in iron-deplete conditions. A) Gc strain FA1090 Opaless 130 was inoculated onto IL8-treated, adherent human PMNs or into media without PMNs, and incubated for 1 h. PMNs were treated with saponin, and Gc were collected and processed for Western blotting. Gc lysates were separated by 10% SDS-PAGE, transferred to a nitrocellulose membrane, and stained with rabbit anti-TbpB polyclonal antisera. The intensity of TbpB is reported relative to the loading control Zwf, which was recognized with rabbit anti-Zwf antiserum. A representative blot from a single experiment is shown. B) Quantification of TbpB/Zwf ratio from 3 independent experiments, using PMNs from 3 unrelated individuals. Error bars indicate SEM. Significance was determined by one-way ANOVA with Holm–Sidak correction for multiple comparisons, * indicates p < 0.05, ** p < 0.01 and *** p < 0.001. C and D) Strain FA1090 Opaless 130 was iron starved in media containing 10 μM deferoxamine (DFO) ± 10 μM Fe(NO_3_)_3_ for 2.5 hours then was inoculated onto IL8-treated, adherent human PMNs or into media without PMNs. Percent Gc survival was calculated by enumerating colony-forming units (CFU) from PMN lysates or media control at 3h post-infection as the percent of CFU for that strain at 0 min. Shapes are replicates from different donors’ PMNs. *n* = 4 independent experiments. C) Significance was determined by two-way ANOVA with Holm–Sidak correction for multiple comparisons. ** indicates *p* < 0.005. ns indicates not significant. D) Data is reported as the change in percent survival for Gc exposed to PMNs compared to Gc in media without PMNs from panel C. Significance was determined by one-way ANOVA with Holm–Sidak correction for multiple comparisons. * indicates *p* < 0.05.

38 of the 413 total PMN-responsive DE genes identified in either Gc strain were associated with iron acquisition (**Fig 3** and **S3 Dataset**). These results led us to hypothesize that Gc responds to PMN challenge by increasing expression of iron acquisition proteins to overcome human nutritional immunity. As a readout of the iron status of Gc, we analyzed the TonB-dependent transferrin receptor TbpAB (NGO1495-NGO1496), which is required for symptomatic infection in male urethral challenge and shows Fur-dependent repression of expression in high iron conditions [28,35]. TbpB expression was induced in the infection medium (mono-culture) after 1h compared to Gc at 0h. Induction of metal acquisition genes in the Gc_1h condition may be explained by the lack of accessible iron for Gc in the infection medium (RPMI+10% FBS), as Gc TbpAB can only use human transferrin, not bovine [36,37]. In line with decreases in iron-responsive transcripts observed by RNA-seq, TbpB protein levels in FA1090 Opaless significantly decreased after 1h of PMN infection compared to mono-cultured Gc (**Fig 4A and 4B**). Dampening of TbpB protein expression during exposure to PMNs suggests that nutrient metals, specifically iron, are more available and/or Gc requirements for metals are lower following Gc co-culture with PMNs compared with unexposed Gc. We then evaluated survival of Gc pre-incubated with deferoxamine (DFO) to induce iron starvation upon exposure to PMNs. In iron replete conditions, Gc survived better in the absence of PMNs than in the presence of PMNs (**Fig 4C and 4D**). However, iron-starved Gc survived significantly better in the presence of PMNs than in their absence (**Fig 4C and 4D**). This phenotype was reverted in the presence of Fe(NO_3_)_3_, indicating that the effect of DFO is iron specific. These data suggest that PMNs unexpectedly support Gc survival in iron limited conditions. In total, these results show a coordinated transcriptional response of Gc towards PMNs, particularly in oxidative stress responses and nutrient acquisition.

### PMNs upregulate select gene subsets in response to Gc infection

To complement the analysis of Gc responses to PMNs, we examined the PMN responses to Gc across three PMN donors (**S4 Dataset**). Using Ingenuity Pathway Analysis (IPA, Qiagen) and selecting for human genes that were 2-fold or more DE in infected PMNs compared to uninfected PMNs at 1 h (Gc+PMN_1h vs PMN_1h), we identified IPA Disease and Functions that were shared across the conditions or unique to specific exposure conditions. From our initial lists of all tested genes expressed under either infection condition (FA1090 Opaless exposed or H041 exposed) (**S4 Dataset**) IPA mapped 358 and 271 genes, respectively, that met 2-fold DE cutoffs using the ‘neutrophil’ cell specific IPA parameter.

**Fig 5:**
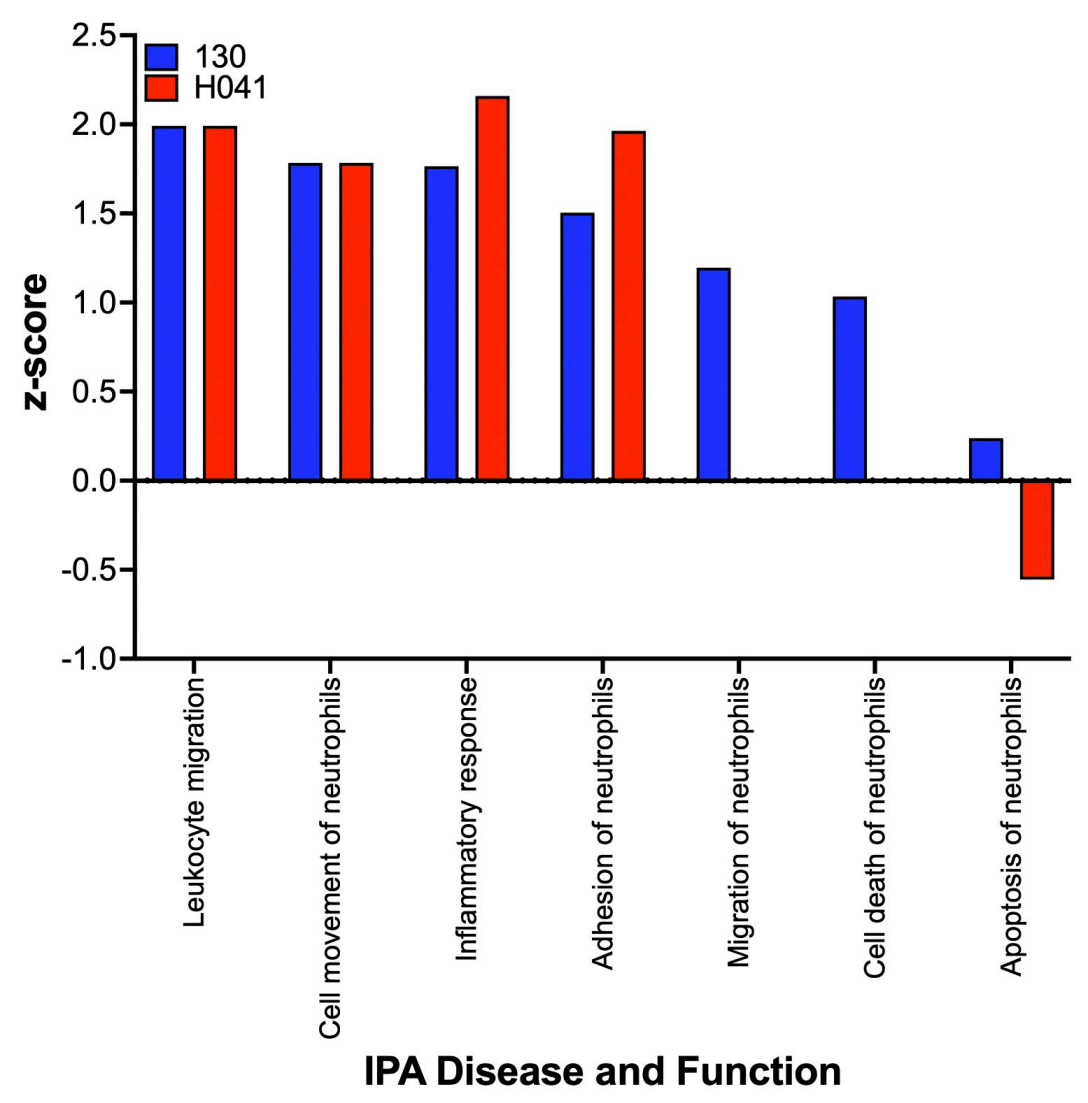
Ingenuity Pathway Analysis (IPA)-enriched Disease and Functions. Utilizing neutrophil cell specific parameters, resulting functions of genes DE in Gc-PMN co-cultures vs PMN mono-cultures at 1h are shown. Blue: co-culture with FA1090 Opaless 130; red: H041. The IPA determined Z-scores of Disease and Function enrichment by DE genes are shown (>0 reveals activation, while <0 reveals inhibition).

The ‘neutrophil’ cell specific IPA Disease and Functions categories enriched were broadly associated with neutrophil immune responses and inflammatory processes (**Figs 5 and S10**). Categories that IPA identified as overrepresented in PMNs infected by both strains were ‘leukocyte migration’, cell movement of neutrophils’, ‘inflammatory response’ and ‘adhesion of neutrophils’, accounting for over 50% of the regulated functions (**Fig 5**). All four of these processes have previously been shown to be associated with bacterial infections [38–41]. Functions ‘migration of neutrophils’ and ‘cell death of neutrophils’ were activated (positive Z-score) only for FA1090 Opaless infected PMNs, whereas ‘apoptosis of neutrophils’ was activated for FA1090 Opaless infected PMNs but inhibited (negative Z-score) in H041 infected PMNs (**Fig 5**). Gc have previously been shown to delay the spontaneous apoptosis of PMNs [18–20].

We also analyzed PMN DE genes using the ’immune cells’ specific IPA parameter under the rationale that there are a limited number of available datasets for PMNs within the IPA databases, and that there is a small (<5%) fraction of non-neutrophil immune cells in our PMN preparations. The broader ‘immune cells’ parameter successfully mapped 873 and 621 genes for FA1090 Opaless exposed and H041 exposed PMNs, respectively (**S10 Fig**). Similar, but less specific Disease and Functions categories were enriched upon exposure to Gc with limited effect of strain background when IPA analysis was broadened to ‘immune cells’ specific parameters. Of the 63 ‘highly modulated’ functions, as determined by a Z-score of at least 2 in either of the conditions, 41% were enriched upon exposure to both strains, while 17% were strain-specific (**S10 Fig**).

#### PMNs upregulate cytokine secretion and migratory responses

**Fig 6:**
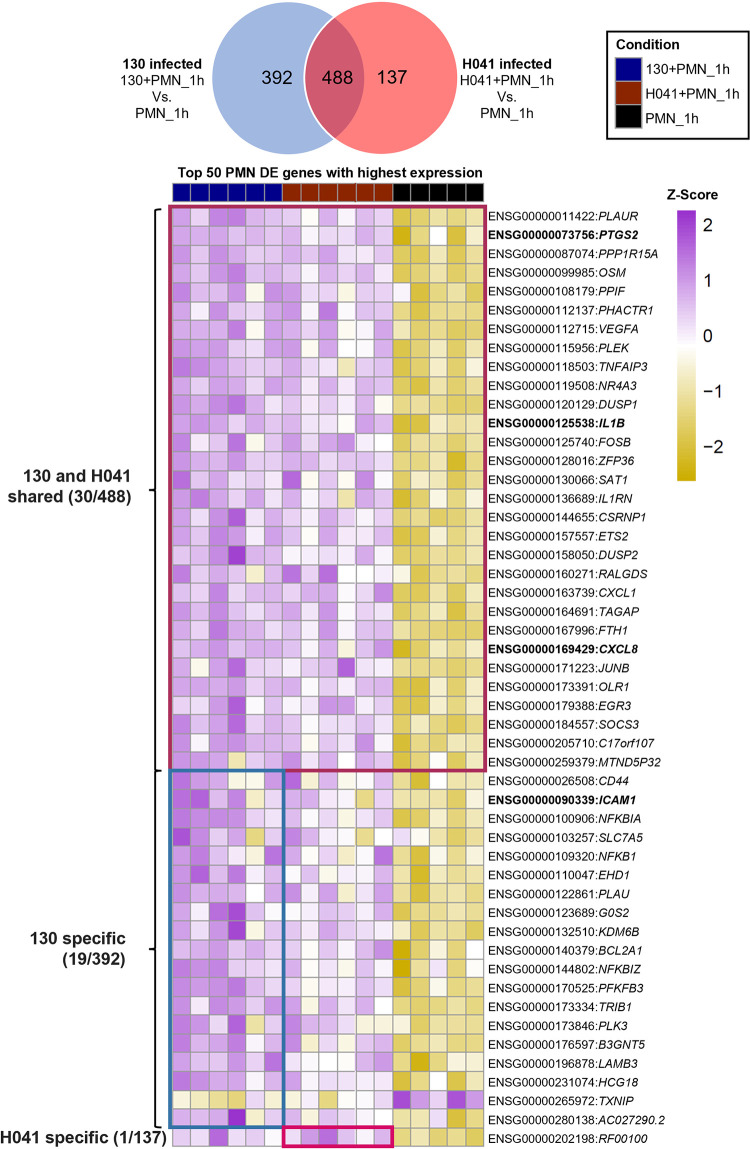
Venn diagram of differentially expressed (DE) PMN genes (Dataset S4) and a Z-scored heatmap of the top 50 PMN genes with the highest expression among DE genes, arranged by infection condition. Highest expression was based on average VST counts across all samples. Boxes on the heatmap correspond to the different Venn intersections to which they belong. Bold and italicized genes were examined in subsequent experiments.

**Fig 7:**
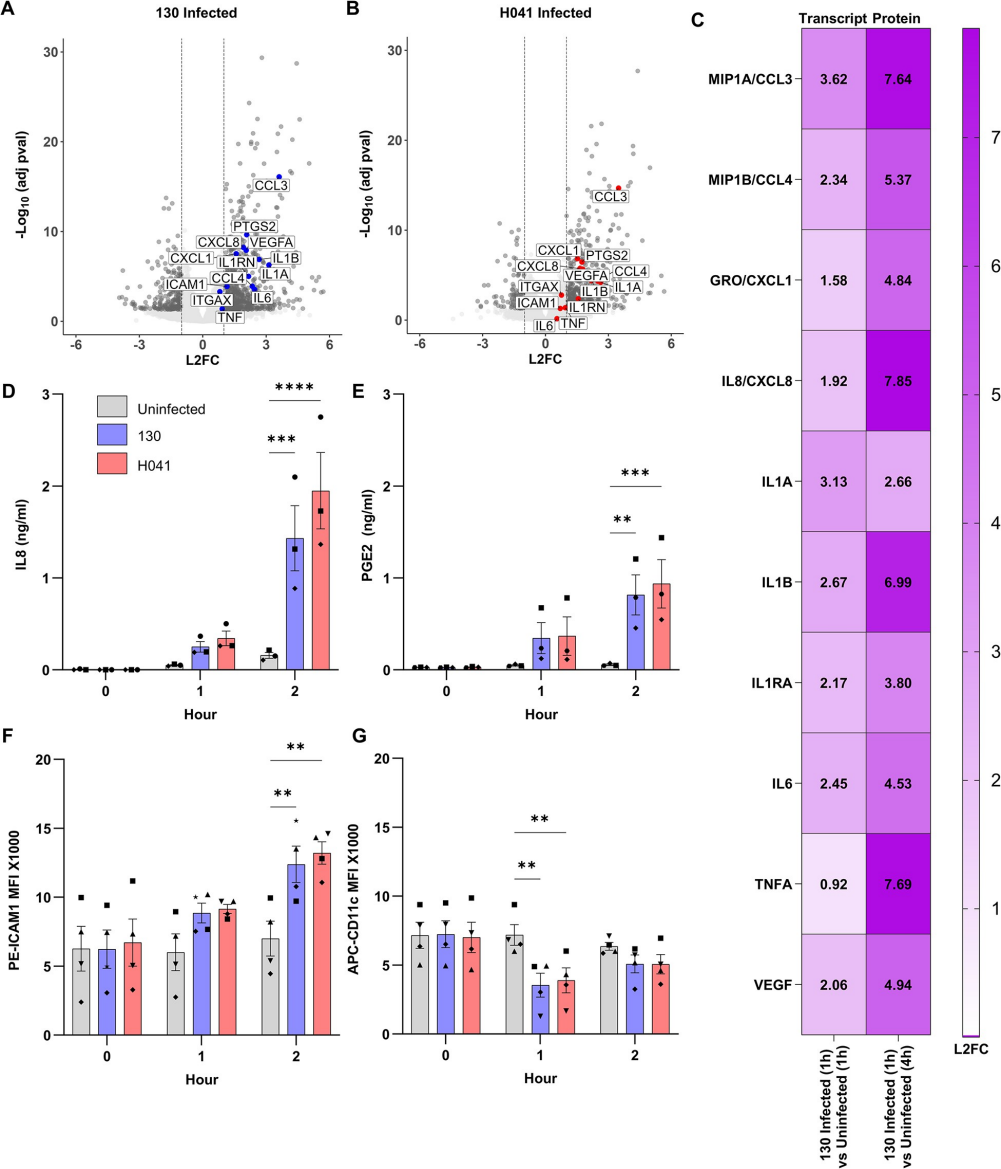
PMNs increase production of pro-inflammatory cytokines and adhesion molecules upon Gc infection. A-B) Scatter plot of Log_2_(Fold Change) (L2FC) of all transcripts vs the -Log_10_(adj pval) for PMNs infected with (A) FA1090 Opaless 130 or (B) H041. Targets that were selected for additional analysis are labeled. C) Supernatants were collected from uninfected and FA1090 Opaless 130-infected adherent PMNs after 4h and quantified by multiplex cytokine analysis (“Protein”). Selected targets identified from PMN DE genes are displayed. The Log_2_(Fold Change) (L2FC) in detected protein for 130 infected PMNs at 4h vs uninfected PMNs at 4h is reported. The corresponding L2FC transcript levels identified by RNA-seq for 130+PMN_1h vs PMN_1h is also indicated (“Transcript”). D and E) Supernatants were collected from PMNs left uninfected or exposed to FA1090 Opaless 130 or H041 at the indicated timepoints. IL8 (D) and PGE2 (E) concentrations were measured by ELISA. Bars represent the mean ± SEM. n = 3 independent experiments. F and G) PMNs were collected and assessed by flow cytometry at the indicated timepoints. ICAM1 (F) and ITGAX (CD11c) (G) surface expression was assessed by Median Fluorescent Intensity (MFI) of PE-ICAM1 and APC-CD11c on CD11b+ /CD14- /CD16+ /CD49- cells (i.e., neutrophils). Bars represent the mean MFI ± SEM. n = 4 independent experiments. D-G) Shapes are data points from different donors’ PMNs. Significance was determined by two-way ANOVA with Holm–Sidak correction for multiple comparisons, *indicates p < 0.05.

While IPA provided a snapshot of the nature of the PMN response with and without Gc, we took the complementary approach of analyzing those genes that were most changed in expression in our experimental conditions. The top 50 most highly expressed DE genes, based on their combined normalized expression [VST counts] across both experimental conditions, were all protein coding (**Fig 6 and S4 Dataset**). Although these are not the most dramatically DE gene products when plotted as -Log_10_(adj pval) against the Log_2_[Fold Change] (L2FC) (**Fig 7A and 7B**), we reasoned that these highly expressed PMN genes likely perform the most robust responses to Gc infection. We performed further experimentation on selected gene products or metabolites from this category that are surface expressed or secreted, with the rationale that these could be investigated in primary human cells that are not genetically manipulatable. Several of these highly expressed DE genes were also common drivers of ‘neutrophil’ and ‘immune cells’ specific category enrichment identified by IPA (**S5 Dataset**). Manual curation based on GeneCards [42] description of these genes indicated that they are mainly involved in regulation of inflammation (*NFKB1*, *NFKBIA*, *NFKBIZ*, *NR4A3*, *EGR3*, *FOSB*, *DUSP1*, *DUSP2*, *JUNB*, *SOCS3*, *ZFP36*), adhesion and migratory responses (*ICAM1*, *CD44*, *FLNA*, *LAMB3*, *PHACTR1*, *PLAUR*), production of cytokines and bioactive lipids (*CXCL1*, *CXCL8*, *PTGS2*, *IL1B*, *IL1RN*, *OSM*, *SAT1*, *VEGFA*), regulation of apoptosis (*CSRNP1*, *BCL2A1*, *ETS2*, *G0S2*, *TNFAIP3*, *PPIF*, *PPP1R15A*, *PFKFB3*), protein ubiquitination (*EHD1*, *RALGDS*, *TAGAP*), signaling (*PLEK*, *PLK3*, *TRIB1*, *OLR1*), regulatory RNAs (*HCG18*, *RF00100*), serine protease (*PLAU*), production of reactive oxygen species (*TXNIP*), metabolite transport (*SLC7A5*), metal homeostasis (*FTH1*), glycolypid modification (*B3GNT5*), chromatin remodeling (*KDM6B*), and transcripts of unknown functions (*C17orf107*, *MTND5P32*, *AC027290*.*2*).

To characterize the inflammatory environment during Gc-PMN co-culture, we conducted multiplexed cytokine profiling of Gc and PMNs at 4h post-infection, to account for de novo transcription and translation and allow for accumulation of cytokines in the supernatant (**Fig 7C**). Upregulated cytokine transcripts from RNA-seq (**Fig 7A–7C**) correlated with increased release of cytokines. Relative to the uninfected control, Gc-infected PMNs released over 4 times (L2FC > 2) the levels of IL1A, IL1B, IL1RA, IL6, IL8, MIP1A (CCL3), MIP1B (CCL4), CXCL1 (GRO), TNFA, and VEGFA (**Fig 7C**). These results imply the ability of PMNs to coordinate the robust recruitment of more PMNs and other leukocytes during Gc infection. *CXCL8* (*IL8*) was the most highly transcribed DE gene by VST count in all Gc infected PMN conditions. In PMNs, IL8 induces upregulation of adhesion factors, priming of the oxidative burst, and release of lysosomal enzymes; and PMNs have long been known to produce IL8 upon stimulation with inflammatory mediators such as lipopolysaccharide [43]. Increased levels of IL8 are found in secretions of men urethrally challenged with Gc but were postulated to be produced locally by the infected mucosa, rather than by infiltrating leukocytes [16]. Since IL8 was near the upper limit of detection by cytokine array (**Fig 7C**), we measured concentrations of IL8 released from infected and uninfected PMNs by ELISA (**Fig 7D**). Here, IL8 pretreatment of PMNs was omitted to avoid confounding the detection of newly released IL8. Concentrations of IL8 released from PMNs significantly increased during infection with both Gc strains but not in uninfected PMNs, beginning at 1h and most pronounced at 2h post-infection (**Fig 7D**). Thus, PMNs respond to Gc by releasing IL8, which is predicted to recruit and activate additional PMNs at the site of infection.

In addition to *CXCL8* and *IL1B*, we also observed multiple genes previously shown to be involved in responses to gonococcal infections, including *PTGS2* and *ICAM1*. *PTGS2* (Prostaglandin-Endoperoxide Synthase 2, COX-2) was found to only be enriched in processes from Gc exposed PMNs when using IPA’s ‘immune cells’ parameters (**S5 Dataset**). PTGS2 is induced during inflammation and synthesizes Prostaglandin H, which is highly unstable [44]. Prostaglandin H is rapidly converted to Prostaglandin E2 (PGE2) by the constitutively expressed Prostaglandin E Synthase 2, and PGE2 production switches PMNs to an anti-inflammatory state to promote inflammation resolution [44,45]. By ELISA, the concentration of PGE2 released by PMNs significantly increased following infection with both Gc strains, but not uninfected PMNs, beginning at 1h and increasing at 2h post-infection (**Fig 7E**). These findings suggest that production of PGE2 modulates the outcome of inflammation at sites of Gc infection, a hypothesis to be tested in future studies.

The integrin *ICAM1*, encoding an adhesion factor involved in signaling that was upregulated in Gc exposed PMNs, was targeted for additional analysis (**S5 Dataset**). ICAM1 is not considered a conventional PMN protein, yet it was transcribed at high levels (**S4 Dataset**). ICAM1 is canonically expressed on endothelial cells to enable the extravasation of leukocytes from the blood into tissues but is also expressed by PMNs, where it modulates effector functions such as ROS production and phagocytosis [46,47]. We assessed whether ICAM1 was truly upregulated by neutrophils upon infection with Gc or was upregulated due to the presence of other cell types present at low levels in our purified PMN population. ICAM1 surface protein expression on PMNs following exposure to both strains of Gc at 1 and 2h post-infection was measured by flow cytometry (**Fig 7F**). ICAM1 was expressed on purified human neutrophils (CD11b^+^/CD14^-^/CD16^+^/CD49^-^ cells) and surface levels of ICAM1 were significantly increased on neutrophils following Gc exposure (**Fig 7F**).

As a control, we also assessed *ITGAX* (*CD11c*) was not significantly DE in infected PMNs. ITGAX is primarily considered a dendritic cell marker; however, PMNs can acquire antigen-presenting capabilities, including the induced expression of ITGAX, upon stimulation with select cytokines [48]. We recently reported CD11c was present at low levels on resting PMNs [49]. As with ICAM1, CD11c surface protein expression on PMNs following exposure to both strains of Gc at 1 and 2h post-infection was measured by flow cytometry (**Fig 7G**). CD11c was expressed on purified human neutrophils (CD11b^+^/CD14^-^/CD16^+^/CD49^-^ cells). Aligning with our RNA-seq data, ITGAX surface expression was not increased in infected PMNs compared to uninfected PMNs, although surface detection of CD11c was significantly decreased at 1h post-infection (**Fig 7G**), suggesting it may be internalized during infection with Gc, possibly during phagocytosis.

Overall, PMN DE genes indicate PMNs mount a concerted transcriptional response to Gc infection, resulting in upregulation of surface and secreted proteins that mediate neutrophil chemotaxis, phagocytosis, and resolution of inflammation. These analyses also reveal previously unidentified pathways that are altered during Gc infection of PMNs and warrant future investigation.

## Discussion

Gonorrhea is characterized by an overt PMN-rich inflammatory response, but despite PMNs’ potent antimicrobial activities, viable Gc are recovered from PMN-rich exudates. The mechanisms behind survival of Gc in this hostile environment have not been fully elucidated. Using dual RNA-seq profiling of Gc and human primary PMNs in a reductive model of acute infection, we performed a comprehensive analysis of the early shared transcriptional response across three unrelated individuals and two isolates of Gc. We discovered that the Gc transcriptional response to PMNs has features in common with the transcriptional response to nutrient metals and oxidative stress, which enhance Gc resistance to hydrogen peroxide and PMNs. Simultaneously, we identified host inflammatory and migratory responses as central to the host transcriptional response against Gc. These findings inform the complex ways that Gc and PMNs respond to one another during co-culture and reveal targets for future mechanistic studies and potential therapeutic intervention.

The transcriptome analyses in this study were conducted using two different bacterial genetic backgrounds: FA1090 (isolated in the 1980s and used extensively in lab experimentation, the background for Opaless clone 130) and H041 (reported in 2011 as a multidrug-resistant isolate) [10,24]. Analysis of major differences between H041 from historic strains, like FA1090, has focused on impacts to antibiotic resistance. Acquisition of enhanced antibiotic resistance in H041 is mainly driven by mutation in regulatory elements (the *mtrR* promoter), point mutations (*penB*) or mosaic alleles (*penA*) [50]. The impact of the strain-specific accessory genes and additional mutations throughout the genome in H041 has not been elucidated. PMNs responded similarly towards Gc regardless of strain background (**Fig 1B**). The Gc response to PMN exposure was mostly shared between the two strain backgrounds (**Fig 1A, PC1**), with some of the differences attributed to fold-changes that did not reach statistical significance but were biologically in the same direction (**Figs 2 and S6 and S7**). For this reason, we focused on the core response of Gc to PMNs in this study, but did note differences between strain backgrounds (**Fig 1A and PC2**). These differences may be partially explained by the de-repression of the *mtrR* regulon in H041 (**Fig 3** and **S3 Dataset**), which harbors a mutation in the promoter for the *mtrR* repressor [10]. Additionally, we noted that 26 Gc DE genes in our dataset were within the regulon of the ModA13 DNA methyltransferase (**Fig 3**). Phase variation of DNA methyltransferases has been shown to differentially regulate several gene sets termed the “phasevarion” [51]. Future studies will mine these data to fully examine strain-specific transcriptional responses during interactions with PMNs and the underlying mechanisms, including the contribution of these and other master regulators.

Of the core Gc response to PMNs, the differentially expressed genes were predominantly involved in metabolism and nutrient acquisition, in categories including central metabolism, amino acid synthesis, cofactor synthesis, and metal acquisition. These changes indicate, in part, growth in a less rich medium for Gc, RPMI + 10% FBS (1h). We recently reported that Gc grows more slowly in RPMI than in GCBL, a rich medium used for culturing Gc [27]. Here, these transcriptional responses revealed Gc metabolic programs specific to culture with PMNs [27]. The transcriptional response reported here differed substantially from the previously reported response of Gc strain 1291 to PMNs in suspension, which showed only 33 genes identified as DE between 10 and 180 min of incubation with PMNs, and 61 DE genes between 10 and 320 min of incubation with PMNs [31] (**S3 Dataset**). Twenty-one of these previously identified genes were DE in Gc upon exposure to adherent PMNs after 60 min in our study (**Fig 3**). This difference may reflect the enhanced ability of adherent PMNs to respond to Gc compared to PMNs in suspension, which are poorly activated and unable to internalize unopsonized, Opa^-^ Gc [18,23]. Additional differences could be due to strain background, media, or timepoints.

Exposure of Gc to PMNs induced a prominent iron response, representing over 13% of the DE genes identified (**Fig 2**). We interpret this response to reflect iron limitation in Gc in the infection conditions used here. Many of the PMN-responsive DE genes associated with iron acquisition are regulated by *fur*, where repression is relieved in low iron conditions [28,52–54]. We used expression of TbpB, which is *fur* regulated, as a readout for the iron status of Gc during infection. While TbpB protein expression was lowest in the inoculated Gc (Gc+PMN_0h), after 1h there was more expression in unexposed Gc (Gc_1h) compared with Gc exposed to PMNs (Gc+PMN_1h). Together, these data suggest that iron limitation is a major mediator of Gc transcriptional responses and resulting protein production in the presence of PMNs. Moreover, the survival defect of iron starved Gc was partially rescued in the presence of PMNs. This surprising finding implies that PMNs may aid in Gc metal acquisition during infection. To our knowledge, this is the first evidence suggesting that Gc directly obtains iron from PMNs to overcome human nutritional immunity. The mechanisms underlying iron-dependent Gc survival from PMNs will be the focus of future research.

Despite Gc encoding numerous oxidative stress defenses, only three dedicated oxidative stress resistance genes in Gc were upregulated in response to PMNs: NGO2059 (*msrA/B*), NGO1769 (*ccpR*) and NGO0602 (*nmlR*) [20]. Instead, many of the DE genes have secondary roles in oxidative stress resistance, including respiration (NGO0805-*cybP*, NGO1276-*aniA*, NGO1328-*cycB*, NGO1371-1374-*ccoPON*, NGO2029-*petA*, and NGO2031-*petC*) metabolite transporters for nutrient metals (NGO0952-*tdfH*, NGO1205 –*tdfJ*, NGO2090-*fecCD*), transporters for cysteine, sulfite/cystine, and sulfate (NGO2011-2013, NGO0372-0374, NGO0446-*cysW*), and cysteine synthesis (NGO0340-*cysK*). The upregulation of genes with secondary roles in oxidative stress resistance is expected, as there is substantial overlap in the Gc transcriptional response to PMNs, iron limitation, H_2_O_2_, and anaerobic transcriptomes [30]. The biological importance of this transcriptional response is in our finding that exposure to PMNs enhanced the resistance of Gc to H_2_O_2_. We note that the FA1090 Opaless isolate used here does not elicit ROS production by PMNs [24] (**S2 Fig**). Moreover, antioxidant gene products are dispensable for Gc to survive exposure to PMNs [55]. Gc exposed to PMNs may sense the hostile host environment and respond by increasing expression of this cohort of genes, regardless of experiencing oxidative stress directly. Alternatively, this response may instead reflect that Gc exposed to PMNs have an enhanced need for nutrients that also resist and repair oxidative damage. Future studies with mutants in these transport/metabolism systems will help to discriminate among these possibilities.

Historically, human PMNs have been considered transcriptionally limited compared to other immune cell types; thus, we hypothesized that any DE human transcripts were likely to be important in the context of Gc infection [56,57]. We observed that the expression of PMN transcripts had shifted towards upregulation, rather than downregulation (**Fig 7A and 7B**), supporting our initial hypothesis. Our results detail a response of PMNs to Gc that is primarily independent of donor and bacterial strain background. We measured upregulation of chemotactic and immune-modulatory gene products in response to Gc; in particular, increased release of the proinflammatory cytokines CXCL8 (IL8), IL1A, IL1B, IL1RA, MIP1A (CCL3), MIP1B (CCL4), and CXCL1 (GRO), and increased surface exposure of ICAM1. These factors are associated with pro-inflammatory pathways and confirm that the core response of PMNs to Gc includes an inflammatory component [58]. Yet we also observed upregulation of *PTGS2* and increased release of its product PGE2, which contribute to inflammation resolution [45]. These results underscore the complexity of the PMN immunomodulatory response to Gc.

Our findings align with a previous report on the inflammatory response of murine PMNs to Gc [59]. PMNs from CEACAM-humanized mice infected with Gc exhibited upregulation of proinflammatory pathways and genes, including *TNFA*, *IL1A*, *CXCL1 (GROα/KC)*, *MIP1A*, *MIP1B*, *PTGS2 (COX-2)*, *NLRP3*, *and OSM* [59]. Production of pro-inflammatory cytokines by mouse PMNs was enhanced by Opa/CEACAM interactions (Opa^+^ Gc and CEACAM-transgenic PMNs), which was confirmed with human PMNs infected with Opa^+^ vs. Opa^-^ Gc. These results suggest that the proinflammatory responses observed with Opa^-^ Gc may be enhanced during infection with Opa^+^ Gc, and may explain some of the differences observed between PMNs exposed to the Opaless FA1090 Gc background compared to H041, which harbors native *opa* loci. Similarly, while the ‘apoptosis of neutrophils’ function was enriched by IPA analysis with a negative Z-score for PMNs co-cultured with H041 Gc, suggesting downregulation of this pathway, it was enriched with a positive z-score for PMNs co-cultured with FA1090 Opaless, suggesting upregulation (**Fig 5**). Gc have previously been shown to delay the spontaneous apoptosis of PMNs [18–20]. Many primary isolates of Gc have been observed to express Opa proteins that bind to CEACAM1 but not CEACAM3 [60] leading to the reduced likelihood of a potent neutrophil response and subsequent killing [18]. Differences in strain backgrounds, as well as Opa expression, may explain the opposing apoptotic process response in PMNs exposed to FA1090 Opaless compared with H041.

In silico IPA functional analyses were conducted using both the specific ‘neutrophil’ cell type category and the broader category of ‘immune cells’. Both were selected because to date there is limited knowledge of the transcriptional pathways directly associated with human neutrophils, some of which may not be curated yet in IPA, while the ‘immune cells’ analysis category may be biased towards more transcriptionally robust cells like macrophages and monocytes. Despite these differences, both analyses generally supported known features of the PMN response to Gc. Some genes drove enrichment of multiple Disease and Functions processes. For instance, *CXCL8* was involved in all the neutrophil-specific Functions that were significant and observed only in PMNs exposed to Gc. Since differential expression calculations for lowly expressed genes are unreliable and require careful validation, we placed additional focus on the top 50 most highly expressed genes that were also DE. A strength of this approach is that previously unappreciated PMN functions were revealed, for instance the appearance of *ICAM1* as a candidate PMN response gene, which is less likely to be categorized as a neutrophil marker in pathway analysis databases such as IPA. The importance of PMNs in diverse inflammatory and infection conditions warrants reworking of available analytical pipelines to better assess the impact of atypical transcripts for these critical cell types.

At the single time point examined, we observed some limited evidence of potential transcriptional crosstalk between Gc and PMNs. For example, we observed the upregulation of Gc metal acquisition genes, and simultaneously identified corresponding host transcripts for manipulation of metal homeostasis and trafficking for nutritional immunity including *FTH1* (ferritin heavy chain) [61]. However, we did not identify changes in transcripts for metal sequestering proteins like lactoferrin and calprotectin (S100A8/S100A9), which are highly abundant as pre-formed proteins within PMNs and have well-established functions in metal sequestration during Gc infection [18,62–67]. Despite this fact, we did observe increases in expression of the TDT *tdfH*, which we previously found can pirate zinc directly from calprotectin to subvert nutritional immunity [66]. At this early time point, PMNs may rely more on post-translational activities like degranulation rather than transcriptional regulation to restrict metals from invading pathogens, including Gc. Going forward, the transcriptional data gathered here needs to be integrated with post-translational activities like regulated secretion and activity of antimicrobial enzymes to understand the overall PMN response to Gc. Alternatively, the limited transcriptional crosstalk observed may indicate that the initial response of PMNs to Gc is nonspecific and proinflammatory for host defense (**Fig 5**). The ability to circumvent both broad and specific PMN antimicrobial responses through unique transcriptional programs mark Gc as a successful pathogen.

Our analysis using multiple human donors and two genetically and phenotypically different isolates of Gc allowed us to identify a core response to infection in both Gc and PMNs. The dual transcriptomes of Gc and human PMNs generated here provide an unbiased foundation for subsequent investigations into host-pathogen interactions during PMN challenge and are a critical reference to support further human infection studies by microbial pathogenesis research communities. Our study highlights nutritional immunity and modulation of inflammation as key features of the Gc-PMN interface. These findings reveal new bacterial and host targets for antimicrobial therapy, vaccine design, and prevention of inflammatory damage in the context of drug-resistant gonorrhea.

## Materials and methods (See S1 Text for additional details)

### Ethics statement

Human Subjects: Venous blood was collected from adult healthy human subjects with their signed informed consent. All materials collected were in accordance with a protocol (#13909) approved by the University of Virginia Institutional Review Board for Health Sciences Research.

### Bacterial strains and growth conditions

Gc strain FA1090 Opaless clone 130 is a non-variable Opa-deficient (Opa^-^) derivative of the FA1090 background constitutively expressing the pilin variant 1-81-S2 [24]. FA1090 constitutively expressing OpaD (Opa^+^) is isogenic in the 130 background [24]. Strain H041 was received from R. Nicholas. Genomic DNA from strain JKD5069 (*ecf*::Ω) [34] from C. Kahler was transformed into the FA1090 Opaless and Opa^+^ background and selected on 40μg/ml kanamycin. Gc was grown on Gonococcal Medium Base (GCB, Difco) plus Kellogg’s supplements [68] at 37°C with 5% CO_2_. For PMN experiments, Gc were grown in liquid medium (GCBL) for successive rounds of dilution to mid-logarithmic phase and enriched for piliation, as previously described [69]. Where indicated, Gc were exposed to 10μM deferoxamine (Sigma) and 10μM Fe(NO_3_)_3_ (Sigma) during log phase growth to induce iron starvation and replenish iron in the medium, respectively. The genomes of strains FA1090 WT, FA1090 Opaless clone 130, and H041 used in this study were sequenced using PacBio technology (NCBI BioProject PRJNA508744, Genbank whole genome shotgun accessions WHPG00000000, WHPI00000000, and WHPH00000000, respectively).

### Gc-PMN co-culture

PMNs were isolated from venous blood as previously described and used within 2h of isolation [22]. Synchronized Gc infection of adherent, IL8 treated PMNs was conducted as previously described [22]. Gc-PMN samples were harvested immediately (Gc+PMN_0h) or after 1h at 37°C with 5% CO_2_ (Gc+PMN_1h), by washing in ice-cold PBS and pooling 6 technical replicates in RNAprotect Cell Reagent (Qiagen) according to manufacturer instructions. Mono-cultures of Gc without PMNs at 1h (Gc_1h) and PMNs without Gc (PMN_0h, PMN_1h) were also collected. Where indicated, CFU were enumerated from PMNs lysed in saponin at specified time points and expressed relative to the CFU at 0h (100%).

### RNA extraction, library construction, and sequencing

Neutrophil granules contain RNases that are released upon cell lysis, complicating the recovery of intact RNA from PMNs [70]. Addition of 20 mM EDTA prior to lysis with the Qiagen RNA extraction kit reduced RNA degradation and improved RNA quality and quantity for the preparation of libraries and subsequent sequencing (**S1 Dataset**). Bacterial and human rRNAs were depleted, 300 bp-insert strand-specific RNA-seq Illumina libraries were constructed, and RNA-seq was conducted on 150 nt pair-end runs using the Illumina HiSeq 4000 platform using two biological replicates for each condition. See **S1 Dataset** for total number of reads generated per sample used in this study. In each sample >80% of reads mapped to either the human or Gc genome (e.g. in a sample, ~30% of reads map to Gc and ~50% to human). No sequences mapping to Gc or human were filtered out or discarded. There were >3.5 million reads mapping to the Gc genome in samples containing bacteria, and rarefaction curves indicate high coverage of both genomes in the datasets (S3 Fig). RNA-seq data generated in this study are deposited at Gene Expression Omnibus (GEO) database under accession number GSE123434.

### Transcriptomic data processing

Illumina reads were trimmed for adaptor sequence and quality. Bacterial reads were mapped to the FA1090 WT genome (GenBank Accession: WHPG00000000) or H041 (GenBank Accession: WHPH00000000) genomes using bowtie v1.0, and human reads were mapped to the 2018 GRCh38 Human genome assembly using HISAT v2.0 [71,72]. On average, ~3.7% of transcripts mapped to rRNA across all samples following rRNA depletion. Opa gene deletions in the FA1090 Opaless clone 130 were verified by absence of RNA-seq read coverage for those loci (**S11 Fig**) in the FA1090 WT genome used for read mapping. For details on RNA-seq data analyses and tools used to generate **Figs 1, 2, 6, S5, S6, and S7**, see **S1 Text** and **Data Availability** below. One replicate of the paired D60 PMN alone samples (PMN_0h/PMN_1h) (Fig 1B, black triangle) was found in preliminary analysis to be mislabeled. To avoid confusion, this replicate was excluded from the study, leaving 5 PMN_1h samples for analysis. All predictions of differentially expressed (DE) genes were performed using DEseq2 and filtered using an FDR cutoff of 0.05 and an absolute Log₂(Fold Change) cutoff of 1 [73]. Gonococcal and human DE genes are listed in **S3** and **S4 Datasets,** respectively.

Bacterial protein coding gene differential expression (DE) estimation was performed by comparing each Gc strain as Gc+PMN_1h vs Gc_1h (**Figs 2**, **S6, S7,** and **S3 Dataset**). DE genes were subjected to manual literature surveys for assignment of functional roles (**Fig 3 and S3 Dataset**).

For PMNs, genes that were DE in Gc+PMN_1h vs PMN_1h for both Gc strains were analyzed. Gene identifiers as well as average Log₂(Fold Change) were used as input for Ingenuity Pathway Analysis (IPA) (Qiagen, Redwood City CA) where IPA-defined core analyses for Disease and Biofunctions were performed using ‘neutrophil’ or ‘immune cells’ specific subsets (QIAGEN Inc., https://digitalinsights.qiagen.com/IPA) [74].

### Quantification of cytokine secretion

Multiplexed cytokine quantification was performed using the 38-plex Human Cytokine/Chemokine Magnetic Bead Panel on the Luminex MAGPIX instrument (Millipore). IL8 was measured with Human IL8/CXCL8 DuoSet ELISA (R&D Biosystems) in PMN supernatants diluted 1:4. PGE2 was measured by Prostaglandin E2 (Highly Sensitive) ELISA (Immuno-Biological Laboratories, Inc.) using undiluted samples.

### Flow cytometry

Surface expression of ICAM1 (CD54) and ITGAX (CD11c) was measured on PMNs (CD49d+/CD16+/CD11b+/CD14-) by flow cytometry using Cytek Northern Lights spectral flow cytometer, and data were analyzed with FCS Express (De Novo Software, Pasadena, CA). Fluorescence minus one (FMO) controls were used to set gates for analysis.

### H_2_O_2_ sensitivity assays

FA1090 Opaless Gc was exposed to adherent, IL8 treated PMNs for 1h as described above. PMNs were lysed in 1% saponin, and Gc were pelleted, washed, and resuspended in GCBL at 10^7^ CFU/ml to yield the pre-exposed bacterial population. The concentration of Gc was based on the expected recovery of viable bacteria (**S1 Fig**) and was verified by CFU enumeration. Pre-exposed and naïve (GCBL-grown) Gc were exposed to the indicated concentrations of H_2_O_2_ (Sigma) in GCBL and incubated with rotation for 15 min at 37°C. Bacteria were then immediately diluted into GCBL containing bovine catalase (final concentration of 10 μg/ml; Sigma) to degrade residual H_2_O_2_ and plated at limiting dilution on GCB agar. CFU/mL was derived from enumerated colonies, and percent viability was calculated by dividing CFU/mL at each time point by CFU/mL at 0 min. Significance determined by linear mixed effects model: Survival ~ Treatment + (1 | Subject) + H_2_O_2_ Concentration.

### TbpB expression

Gc were exposed to adherent, IL8 treated PMNs for 1h as described above. PMNs were lysed in 1% saponin, and Gc cells were pelleted (5 min, 3000xg), washed in GCBL, and resuspended in 1X Laemmli sample buffer containing SDS and β-mercaptoethanol. Gc lysates were separated by 10% SDS-PAGE, transferred to nitrocellulose, and blotted for TbpB and Zwf as loading control [75,76]. Quantification of band intensity using infrared secondary antibodies and normalization was performed in ImageStudio Ver 5.2 (LI-COR).

## Supporting information

S1 Text Supplemental Materials and Methods, Description of Datasets, and References. (PDF)

S1 Fig Purity of neutrophils in the human PMN preparation by flow cytometry. (PDF)

S2 Fig Gc experiences an outgrowth period at 1 hour following exposure to PMNs. (PDF)

S3 Fig Rarefaction curves of RNA-seq reads mapped to Gc and Human genes. (PDF)

S4 Fig Venn diagram of PanOCT predicted Gc gene orthologs. (PDF)

S5 Fig Principal Component Analyses (PCA) of Gc and PMN transcriptomes including 0-hour samples. (PDF)

S6 Fig Heatmaps of Gc regulons enriched by FA1090 Opaless 130 specific DE genes. (PDF)

S7 Fig Heatmaps of Gc regulons enriched by H041 specific DE genes. (PDF)

S8 Fig Ecf does not contribute to survival of Gc from PMNs. (PDF)

S9 Fig Gc resistance to hydrogen peroxide is enhanced by exposure to PMNs. (PDF)

S10 Fig Ingenuity Pathway Analysis (IPA)-enriched Disease and Functions. (PDF)

S11 Fig RNA-Seq coverage of Opa gene deletions for FA1090 Opaless 130 strain seen in Integrated Genomics Viewer (IGV) on an FA1090 WT background. (PDF)

S1 Dataset Predicted Gc gene orthologs and RNA-seq mapping statistics for Gc and PMNs. (XLSX)

S2 Dataset Raw and normalized (VST) RNA-seq read counts for Gc and PMNs. (XLSX)

S3 Dataset Bacterial gene log2(fold change) [L2FC] and significance for differentially expressed (DE) genes in FA1090 Opaless 130 and H041 identified. (XLSX)

S4 Dataset Human gene log2(fold change) [L2FC] and significance for differentially expressed (DE) genes identified. (XLSX)

S5 Dataset Ingenuity Pathway Analysis (IPA) Disease and Functions and genes contributing to their enrichment. (XLSX)
