# Supplementary material for: Dual species transcriptomics reveals conserved metabolic and immunologic processes in interactions between human neutrophils and Neisseria gonorrhoeae
Source: PLoS Pathog. 2024 Jul 8;20(7):e1012369. doi: 10.1371/journal.ppat.1012369 (PMC11257400; doi:10.1371/journal.ppat.1012369)
Supplement: S1 Text — (PDF) [file ppat.1012369.s001.pdf]

Supporting Information

Dual species transcriptomics reveals conserved metabolic and immunologic processes in human neutrophil-*Neisseria gonorrhoeae* interactions.

AUTHORS:

Aimee D. Potter*[1], Vonetta L. Edwards*[2], Adonis D'Mello*[2], Mary C. Gray[1], Amol C. Shetty[2], Amy L. Forehand[1], Camille S. Westlake[1], Evan R. Lamb[1], Xuechu Zhao[2], Stephanie A. Ragland[3], Alison K. Criss[#%1], Hervé Tettelin[#%2]

*Co-first authors #Co-senior authors %Co-corresponding

Corresponding: Alison K. Criss

Email: akc2r@virginia.edu

This PDF file includes:

Text S1
Figures S1 to S11
Legends for S1 to S5 Datasets
Supporting References

Other supporting materials for this manuscript, not within this document, include the following:

S1 to S5 Datasets

S1 TEXT

MATERIALS AND METHODS

RNA extraction from PMNs and Gc. Adherent PMNs ($3.6*10^6$ PMNs/well) were treated with 10 nM human IL-8 (R&D Biosystems) in Roswell Park Memorial Institute 1640 medium (RPMI) with 10% fetal bovine serum (FBS) at 37°C with 5% CO_2 for at least 30 min prior to infection. Gc at an MOI of 5 were centrifuged onto PMNs chilled on ice to synchronize infection. Samples were collected in RNAprotect Cell Reagent (Qiagen) at the University of Virginia and shipped on dry ice to the University of Maryland.

After thawing on ice, 20 mM EDTA was added to each sample and incubated at room temperature for 15 min to inactivate RNases released from neutrophils. Samples were pelleted at 13,000 x g for 5 min at 4°C, the supernatant removed, and 100µl of lysis solution added (2.50 U/µl mutanolysin, 4µg/µl Proteinase K, 3µg/µl lysozyme, TE buffer 40µl) plus 4µl EDTA (500 mM). These samples were vortexed and then incubated for 10 min at room temperature with intermittent vortexing every 2 min. Samples were extracted using the Qiagen RNA extraction kit as per manufacturer's recommendations with slight modifications. Briefly, 700µl RLT buffer containing BME (10µl/ml) was added to each sample and vortexed for 30 sec. This was followed by centrifugation for 30 sec at 13,200 x g and the supernatant transferred to a new tube. 800µl 70% ethanol (1 volume based on supernatant volume) was added and 700µl transferred to a RNeasy spin column, spun at 8000 x g for 30 sec and the flow through discarded. This step was repeated until the entire sample passed through the column. 350µl RW1 buffer was then added to the column and spun at 8000 x g for 30 sec, then 80µl of DNase solution (70µl RDD buffer, 10µl DNase) added to each filter and incubated at room temperature for 15 min. 350µl RW1 buffer with 20 mM EDTA was added and incubated for 10 min at room temperature, spun at 8000 x g for 30 sec and the flowthrough discarded. 500µl RPE was added to the column, incubated at room temperature for 5 min, spun at 8000 x g for 1 min and flowthrough discarded. The column was then transferred to a new collection tube, 50µl RNase-DNase free water added

and incubated at room temperature for 5 min followed by a spin at 8000 x g for 1 min and the RNA collected.

To remove any residual DNA, an additional DNase treatment was conducted in solution. 5µl 10X Turbo DNase buffer and 1µl DNA free enzyme (2U/µl) were added to the sample and incubated at 37°C for 30 min. An additional 1µl DNA free enzyme (2U/µl) was added, and samples incubated for an additional 30 min at 37°C. 12µl DNase inactivation reagent was added, and the tube flicked for even distribution. Samples were incubated for 5 min at room temperature with intermittent flicking and centrifuged at 10,000 x g for 2 min. Supernatant (RNA) was transferred to a new tube and stored at -80 °C.

RNA quantification. 1µl of sample or RNA ladder was added to 5µl RNA Tape Station buffer (Agilent; with a maximum buffer concentration of 200 mM Tris, 20 mM EDTA, 50 mM NaCl per sample) as per manufacturer's instructions. Samples were heated to 72°C for 3 min, followed by incubation on ice for 2 min. Samples were analyzed on the Tape Station instrument as per Agilent recommendations and RNA Integrity Numbers (RIN) and RNA concentrations determined (**S1 Dataset**).

Acquisition of high-quality RNA from PMNs. Extraction of high quality and quantity RNA from PMNs is not trivial, as neutrophil granules contain RNases that are released upon cell lysis (1). The standard metric of RNA quality for sequencing (RNA Integrity Number or RIN) is recommended to be over 8 for high quality RNA samples. Conventional RNA isolation from samples containing PMNs gave low quality RNA, with RIN scores as low as 3.6 with concentrations as low as 1.6 ng/µl. We found that addition of 20 mM EDTA for 15 min prior to cell lysis reduced RNA degradation and improved RNA quality and quantity, resulting in samples with average RIN scores of 5.7 for samples containing PMNs, and 9.0 for samples containing Gc alone (**S1 Dataset**). RNA concentrations were also improved with EDTA

treatment, with Gc alone samples averaging 71.4 ng/µl, while those containing PMNs averaged 27.0 ng/µl (**S1 Dataset**). The use of EDTA treatment allowed for RNA of sufficient quality and quantity to be collected for the preparation of libraries and subsequent sequencing.

RNA library construction and sequencing. 300 bp-insert strand-specific RNA-seq Illumina libraries were constructed using RNA that was enriched for mRNA by depletion of ribosomal RNA using the Ribo-Zero rRNA Removal Kits for Gram-negative bacteria and/or for human/mouse/rat (Illumina). RNA that was then fragmented and used for synthesis of strand-specific cDNA using the NEBnext Ultra Directional RNA Library Prep Kit (NEB-E7420L). The cDNA was purified between enzymatic reactions and the size selection of the library performed with AMPure SpriSelect Beads (Beckman Coulter Genomics). The titer and size of the libraries was assessed on the LabChip GX (Perkin Elmer) and with the Library Quantification Kit (Kapa Biosciences). RNA-seq was conducted on 150 nt pair-end runs of the Illumina HiSeq 4000 platform using two biological replicates for each condition.

Transcriptomic data processing. *Neisseria gonorrhoeae*: Sequence reads were trimmed for adaptor sequence and mapped to the FA 1090 WT (GenBank Accession: WHPG00000000) or H041 (GenBank Accession: WHPH00000000) Neisseria genomes using bowtie v1.0 (2) with parameters -mode=v --num_mismatches=2 --file-type=fastq --seedlen=28 --minins=0 --maxins=600 --library-type=fr --args='--sam' --v --gzip .

Homo sapiens: Sequence reads were trimmed for adaptor sequence and mapped to the 2018 GRCh38 Human genome assembly using HISAT v2.0 (3) with parameters -mismatch-penalties=6,2 --softclip-penalties=2,1 --read-gap-penalties=5,3 --ref-gap-penalties=5,3 --min-intronlen=20 --max-intronlen=500000 --score-min=L,0,-0.2 --pen-cansplice=0 --num-threads=1 --pen-noncansplice=12 --pen-canintronlen=G,-8,1 --pen-noncanintronlen=G,-8,1 --rna-

strandness=RF --dta-cufflinks=1 --num-alignments=5 --minins=0 --maxins=600 --no-unal=1 --args=" --v .

Normalized gene expression values and differential gene expression analyses for host and bacterial gene features were calculated as previously described (4). Briefly, gene expression counts for all samples were estimated using HTseq (5). For bacterial genes, counts were then tabularized and counts tables were imported into Rstudio for calculation of differentially expressed (DE) genes (see **Data Availability** in the main document). Rarefaction curves in **S3 Fig** were generated from HTseq counts data (see **Data Availability**). All samples approached plateau, indicating that sequencing depth was sufficient. Principal Component Analyses (PCAs) in **Fig 1 and S5 Fig** were generated using R studio based on normalized Variance Stabilized Transformation (VST) counts acquired using the DESeq2 R package (6). VST counts are transformed gene counts data, that have been normalized for sequencing depth and variance across biological replicates. All predictions of differentially expressed (DE) genes were performed using DEseq2 and filtered using an FDR cutoff of 0.05 and an absolute Log_2(Fold Change) cutoff of 1. DE genes shared between different comparisons or specific to a given comparison were determined using Upset plots (See **Data Availability**). Individual heatmaps of DE genes were generated based on Z-scores of average VST counts per condition (**S2 Dataset**). Gonococcal and human DE genes are listed in **S3 and S4 Datasets**, respectively.

Gc analysis. DE genes were first estimated for the comparisons of 130+PMN_1h vs 130_1h and H041+PMN_1h vs H041_1h using all 130 or H041 genes (**S3 Dataset, Tabs 4-7**). For all Gc+PMN_1h vs Gc_1h conditions, genes that were DE in both 130 and H041, and were also present in FA1090, were determined for each comparison. This revealed 98 core genes (98 genes with only one copy in each Gc strain) that were DE in both strains (**Fig 2**), and 413 core genes DE in at least one strain (**S3 Dataset Tab 1**). The 413 genes were then subjected to a

manual literature survey (7-12) (**Fig 3, S3 Dataset Tab 2**), to reveal differentially regulated gonococcal pathways.

PMN analysis. Unfiltered lists of 15,833 and 15,222 expressed genes from 2 comparisons – 130+PMN_1h vs PMN_1h and H041+PMN_1h vs PMN_1h, respectively – were created and input into Ingenuity Pathway Analysis (IPA) (Qiagen, Redwood City CA). A cutoff of 2-fold (Log_2FoldChange 1 or -1) was implemented and Disease and Biological functions analysis were performed using "neutrophil" or "immune cells" specific parameters (13) (QIAGEN Inc., https://digitalinsights.qiagen.com/IPA, Jan 2024 release). Gene identifiers were linked to their appropriate gene object in the IPA knowledge base and the following specific parameters used: Ingenuity knowledge base genes only; Interactions include endogenous chemicals; All node types; All data sources; Confidence experimentally observed; Species human; Cells_tissue and primary cells_immune cells_granulocytes_neutrophils (referred to as 'neutrophil' in the manuscript) or Cells_tissue and primary cells_immune cells ('immune cells'); All mutations. To confirm that the probability that each biological function, disease or pathway predicted to be involved was not due to chance alone a Fisher's exact test was performed. IPA then computed a score for each network based on the set of genes input which indicate activation or inhibition of the function or pathway (13). From these data, we analyzed Disease and Biological functions with a Z-score cutoff of 2, as this is what IPA evaluates as a statistically significant finding.

Multiplexed cytokine detection (Fig 7). Adherent PMNs were incubated with Gc as described above or left uninfected. PMN supernatants were harvested after 4h of incubation at 37°C with 5% CO_2, then stored at -20°C. PMNs were not pretreated with IL-8 to avoid confounding exogenously added IL-8 and IL-8 released in response to Gc; no differences were noted with or without IL-8 pretreatment in the ability of PMNs to adhere or interact with Gc. Quantification of

secreted cytokines was done by Millipore 38-Plex Human Cytokine/Chemokine Magnetic Bead Panel (HCYTMAG60PMX38BK) on the Luminex MAGPIX instrument and reported as pg/mL.

Enzyme linked immunosorbent assays (Fig 7). Adherent PMNs were incubated with Gc as described above or left uninfected. PMN supernatants were harvested sequentially at baseline (0h) or after 1 or 2h of incubation at 37°C with 5% CO_2, then stored at -80°C. For the IL-8 ELISA, PMNs were not pretreated with IL-8 to avoid confounding exogenously added IL-8 and the IL-8 released in response to Gc; no differences were noted in the ability of PMNs to adhere or interact with Gc. IL-8 was measured by Human IL-8/CXCL8 DuoSet ELISA (R&D Biosystems) with supernatants diluted 1:4. PGE2 was measured by Prostaglandin E2 (Highly Sensitive) ELISA (Immuno-Biological Laboratories, Inc.) using undiluted samples.

Flow cytometry (Fig 7). Adherent, IL-8 treated PMNs were incubated with Gc or left uninfected. PMNs were lifted with 5mM EDTA at 0, 1, and 2h post-incubation. Approximately $1x10^6$ PMNs were washed and stained with PE/Cyanine5 anti-human CD11b Antibody Clone ICRF44 (Biolegend, San Diego, CA) and BV711 Mouse Anti-Human CD14 Clone MφP9 (BD Horizon) to identify polymorphonuclear cells (PMNs) from monocytes, and Brilliant Violet 421 anti-human CD16 Antibody clone 3G8 (Biolegend, San Diego, CA) and PE/Cyanine7 anti-human CD49d Antibody Clone 9F10 (Biolegend, San Diego, CA) to discriminate neutrophils from eosinophils, by flow cytometry. ICAM1 and ITGAX (CD11c) surface expression was assessed by Median Fluorescent Intensity (MFI) of PE anti-human CD54 Antibody clone HCD54 (Biolegend, San Diego, CA) and APC anti-human CD11c Antibody clone 3.9 (Biolegend, San Diego, CA) on $CD11b^+/CD14^-/CD16^+/CD49^-$ cells, i.e. neutrophils. Antibodies were diluted in Brilliant Stain Buffer (BD Horizon) and incubated with samples on ice for 30 min. Samples were also stained with isotype control antibodies to detect non-specific binding. Samples were then resuspended in 2% paraformaldehyde (PFA) until processing by flow cytometry on the Cytek Northern Lights

spectral flow cytometer in the UVA Flow Cytometry Core Facility. Data were analyzed using FCS Express (De Novo Software, Pasadena, CA). Fluorescence minus one (FMO) controls were used to set gates for analysis.

Western blotting (Fig 4). 130 Gc was exposed to adherent, IL-8 treated PMNs for 1h as described above. PMNs were lysed in 1% saponin, and Gc cells were pelleted (5 mins, 3000xg), washed in GCBL, and resuspended in 1X Laemmli sample buffer containing SDS and β-mercaptoethanol. Samples were boiled for 5 minutes and sheared through a 25G needle, then stored at -80ºC. Gc lysates were separated by 10% polyacrylamide SDS-PAGE and transferred onto nitrocellulose. Blots were blocked for 16h in Tris-buffered saline containing 0.05% Tween-20 and 5% nonfat dry milk. TbpB was detected using polyclonal rabbit antisera (a gift of C. Cornelissen, Georgia State University) as described (14), followed by goat anti-rabbit IgG coupled AlexaFluor 680 (Invitrogen). As a loading control, blots were probed with rabbit anti-Zwf (a gift of Aleksandra Sikora, Oregon State University) (15). Quantification of band intensity and normalization was performed in ImageStudio v5.2 (LI-COR).

DATASET LEGENDS

S1 Dataset. Predicted Gc gene orthologs and RNA-seq mapping statistics for Gc and PMNs.
Tab 1: Predicted gene orthologs (including paralogs separated by commas)
Tab 2: RNA-seq mapping statistics
Tab 3: RNA Integrity (RIN) scores

S2 Dataset. Raw and normalized (VST) RNA-seq read counts for Gc and PMNs.
Tab 1: H041 raw read counts
Tab 2: 130 raw read counts
Tab 3: GC core raw read counts
Tab 4: PMN raw read counts
Tab 5: H041 VST read counts
Tab 6: 130 VST read counts
Tab 7: GC core VST read counts
Tab 8: PMN VST read counts
Tab 9: Union of the top 1% of genes affecting PC2, PC3 & PC5 (S5 Fig.)

S3 Dataset. Bacterial gene log_2(fold change) [L2FC] and significance for differentially expressed (DE) genes in FA1090 Opaless 130 and H041 identified from each of the following comparisons.
Tab 1: DE genes in either 130 or H041 for Gc+PMN_1h vs Gc_1h
Tab 2: All DE with corresponding NGO ids compared to select published datasets
Tab 3: DE Genes from selected published datasets
Tab 4: 130+PMN_1h vs 130_1h
Tab 5: H041+PMN_1h vs H041_1h
Tab 6: 130+PMN_1h vs 130+PMN_0h
Tab 7: H041+PMN_1h vs H041+PMN_0h

S4 Dataset. Human gene log_2(fold change) [L2FC] and significance for differentially expressed (DE) genes identified from each of the following comparisons.
Tab 1: Top 50 DE genes with average L2FC Gc+PMN_1h vs PMN_1h sorted by average VST count for Gc+PMN_1h conditions
Tab 2: 130+PMN_1h vs PMN_1h
Tab 3: H041+PMN_1h vs PMN_1h
Tab 4: PMN_1h vs PMN_0h

S5 Dataset: Ingenuity Pathway Analysis (IPA) Disease and Functions and genes contributing to their enrichment.
Tab 1: 130+PMN_1hr vs PMN_1h Disease and Functions enrichment with “neutrophil” cell specific IPA parameters
Tab 2: H041+PMN_1hr vs PMN_1h Disease and Functions enrichment with “neutrophil” cell specific IPA parameters
Tab 3: 130+PMN_1hr vs PMN_1h Disease and Functions enrichment with “immune cells” specific IPA parameters

Tab 4: H041+PMN_1hr vs PMN_1h Disease and Functions enrichment with “immune cells” specific IPA parameters

Tab 5: Gene centric focus of Disease and Functions enriched with “neutrophil” cell specific IPA parameters

Tab 6: Gene centric focus of Disease and Functions enriched with “immune cells” specific IPA parameters

SUPPLEMENTAL REFERENCES

1. Lu L, Li J, Moussaoui M, Boix E. Immune Modulation by Human Secreted RNases at the Extracellular Space. Frontiers in immunology. 2018;9.
2. Langmead B, Trapnell C, M P, Salzberg S. Ultrafast and memory-efficient alignment of short DNA sequences to the human genome. Genome biology. 2009;10(3).
3. Kim D, Langmead B, Salzberg S. HISAT: a fast spliced aligner with low memory requirements. Nature methods. 2015;12(4).
4. Shenoy A, Brissac T, Gilley R, Kumar N, Wang Y, Gonzalez-Juarbe N, et al. *Streptococcus pneumoniae* in the heart subvert the host response through biofilm-mediated resident macrophage killing. PLoS pathogens. 2017;13(8).
5. Anders S, Pyl PT, Huber W. HTSeq--a Python framework to work with high-throughput sequencing data. Bioinformatics (Oxford, England). 2015;31(2).
6. Love M, Huber W, Anders S. Moderated estimation of fold change and dispersion for RNA-seq data with DESeq2. Genome biology. 2014;15(12).
7. Yu C, McClure R, Nudel K, Daou N, Genco C. Characterization of the *Neisseria gonorrhoeae* Iron and Fur Regulatory Network. Journal of bacteriology. 2016;198(16).
8. Quillin S, Hockenberry A, Jewett M, Seifert H. Neisseria gonorrhoeae Exposed to Sublethal Levels of Hydrogen Peroxide Mounts a Complex Transcriptional Response. mSystems. 2018;3(5).
9. Isabella V, Clark V. Deep sequencing-based analysis of the anaerobic stimulon in *Neisseria gonorrhoeae*. BMC genomics. 2011;12.
10. McClure R, Sunkavalli A, Balzano P, Massari P, Cho C, Nauseef W, et al. Global Network Analysis of *Neisseria gonorrhoeae* Identifies Coordination between Pathways, Processes, and Regulators Expressed during Human Infection. mSystems. 2020;5(1).
11. Nudel K, McClure R, Moreau M, Briars E, Abrams AJ, Tjaden B, et al. Transcriptome Analysis of *Neisseria gonorrhoeae* during Natural Infection Reveals Differential Expression of Antibiotic Resistance Determinants between Men and Women. mSphere. 2018;3(3).
12. Folster J, Johnson P, Jackson L, Dhulipali V, Dyer D, Shafer W. MtrR modulates rpoH expression and levels of antimicrobial resistance in *Neisseria gonorrhoeae*. Journal of bacteriology. 2009;191(1).
13. Krämer A, Green J, Pollard J, Tugendreich S. Causal analysis approaches in Ingenuity Pathway Analysis. Bioinformatics (Oxford, England). 2014;30(4).
14. Price G, Masri H, Hollander A, Russell M, Cornelissen C. Gonococcal transferrin binding protein chimeras induce bactericidal and growth inhibitory antibodies in mice. Vaccine. 2007;25(41).
15. Wierzbicki IH, Zielke RA, Korotkov KV, Sikora AE. Functional and structural studies on the *Neisseria gonorrhoeae* GmhA, the first enzyme in the glycero-manno-heptose biosynthesis pathways, demonstrate a critical role in lipooligosaccharide synthesis and gonococcal viability. Microbiologyopen. 2017;6(2).
