# Supplementary material for: Dual species transcriptomics reveals conserved metabolic and immunologic processes in interactions between human neutrophils and Neisseria gonorrhoeae
Source: PLoS Pathog. 2024 Jul 8;20(7):e1012369. doi: 10.1371/journal.ppat.1012369 (PMC11257400; doi:10.1371/journal.ppat.1012369)
Supplement: S1 Fig — (PDF) [file ppat.1012369.s002.pdf]

SUPPLEMENTAL FIGURES

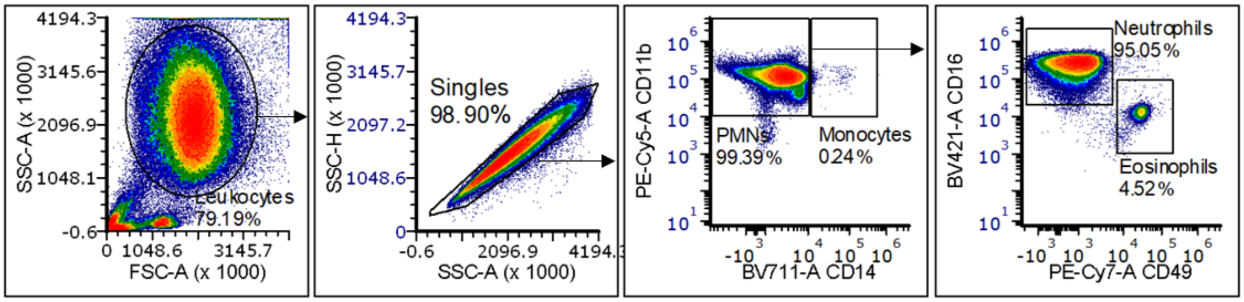

<u>S1 Fig.</u> Purity of neutrophils in the human PMN preparation by flow cytometry. IL-8-treated, adherent human PMNs were left untreated, or exposed to FA1090 Opaless 130 or H041 for 0, 1, or 2h. Cells were subsequently examined by flow cytometry using forward scatter (FSC-A) and side scatter (SSC-A) to identify single leukocytes, by PE-Cy5-CD11b and BV711-CD14 to distinguish polymorphonuclear cells (PMNs) from monocytes, and by BV421-CD16 and PE-Cy7-CD49 to distinguish neutrophils from eosinophils by flow cytometry. PE-ICAM1 and APC-CD11c was also assessed (**Fig 7**). Cytometry plots from one representative preparation of uninfected PMNs at 0h are shown.
