# Supplementary material for: Dual species transcriptomics reveals conserved metabolic and immunologic processes in interactions between human neutrophils and Neisseria gonorrhoeae
Source: PLoS Pathog. 2024 Jul 8;20(7):e1012369. doi: 10.1371/journal.ppat.1012369 (PMC11257400; doi:10.1371/journal.ppat.1012369)
Supplement: S2 Fig — (PDF) [file ppat.1012369.s003.pdf]

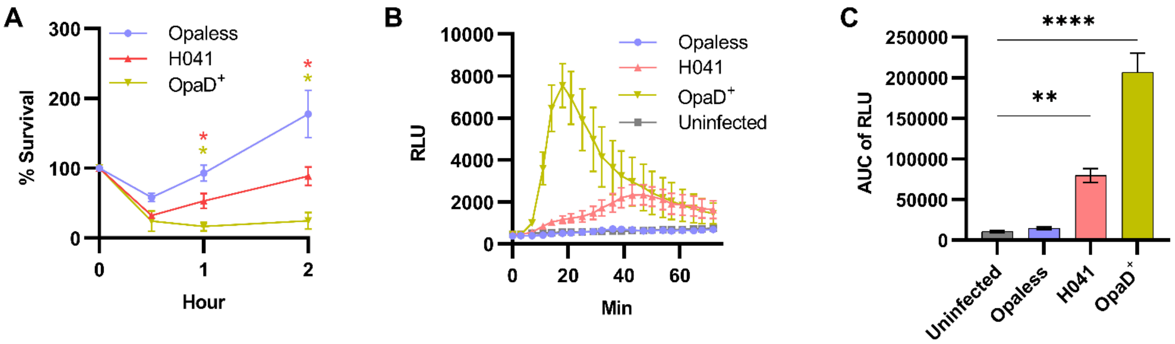

<u>S2 Fig.</u> Gc experiences an outgrowth period at 1 hour following exposure to PMNs. A) Gc strains FA1090 Opaless 130, an isogenic constitutive OpaD expressing strain, and H041 were exposed to adherent, IL-8-treated primary human PMNs. Percent Gc survival was calculated by enumerating colony-forming units (CFU) from PMN lysates at 30, 60, and 120 min and reported as the percent of CFU for that strain at 0 min. Significance was determined by one-way ANOVA with Holm–Sidak correction for multiple comparisons. * indicates $p < 0.05$ compared to 130. n = 4 independent experiments. B) FA1090 Opaless 130, $OpaD^+$ and H041 were exposed to primary human PMNs in suspension in the presence of luminol. ROS production was measured over the course of 60 minutes (min) as the relative light units (RLU) generated by luminol-dependent chemiluminescence. n = 4 independent experiments. C) The area under the curve (AUC) was calculated for (B). Significance was determined by one-way ANOVA with Holm–Sidak correction for multiple comparisons. * indicates $p < 0.0001$ compared to PMNs alone.
