# Supplementary material for: Dual species transcriptomics reveals conserved metabolic and immunologic processes in interactions between human neutrophils and Neisseria gonorrhoeae
Source: PLoS Pathog. 2024 Jul 8;20(7):e1012369. doi: 10.1371/journal.ppat.1012369 (PMC11257400; doi:10.1371/journal.ppat.1012369)
Supplement: S3 Fig — (PDF) [file ppat.1012369.s004.pdf]

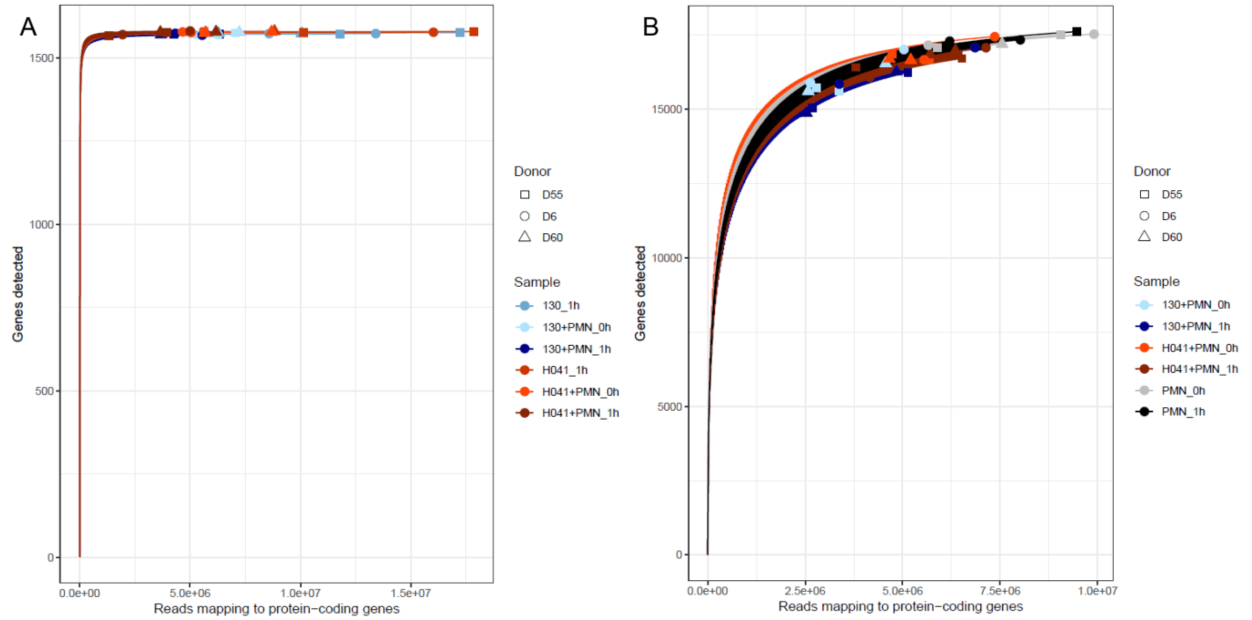

<u>S3 Fig.</u> Rarefaction curves of RNA-seq reads mapped to Gc and Human genes. Samples with curves that plateau have sufficient reads mapped across the respective genome to achieve saturation. A) Rarefaction curves of reads mapped to the Gc core genes (1582 core genes without paralogs, **S1 Dataset**) for samples containing FA1090 Opaless 130 and H041 reads. The minimum required mapped reads to reach saturation for the ~1600 core protein coding genes in Gc was 150,000-200,000. B) Rarefaction curves of reads for all PMN containing samples mapped to human genes. For host PMN transcripts, the minimum required mapped reads to reach saturation for the ~17,500 genes was 2,500,000. The proportion of Gc to total mapped transcripts in infected PMN samples was ~25-35%.
