# Supplementary material for: Dual species transcriptomics reveals conserved metabolic and immunologic processes in interactions between human neutrophils and Neisseria gonorrhoeae
Source: PLoS Pathog. 2024 Jul 8;20(7):e1012369. doi: 10.1371/journal.ppat.1012369 (PMC11257400; doi:10.1371/journal.ppat.1012369)
Supplement: S4 Fig — (PDF) [file ppat.1012369.s005.pdf]

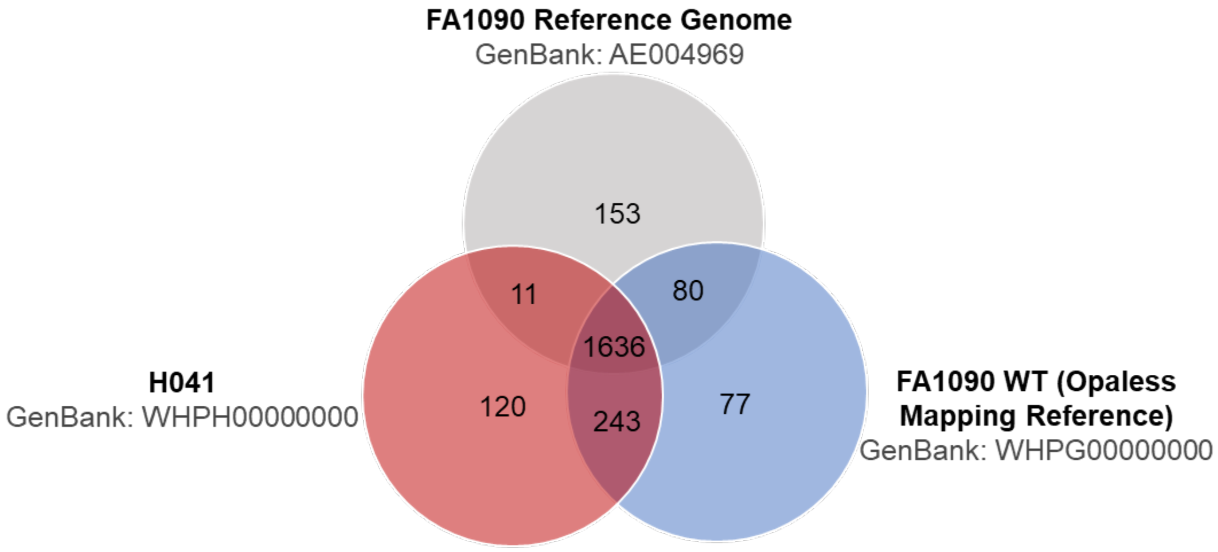

<u>S4 Fig.</u> Venn diagram of PanOCT predicted Gc gene orthologs. Published FA1090 reference genome (Accession: AE004969) in gray, sequenced lab strain FA1090 WT (Accession:WHPG00000000, to which all Opaless 130 samples were mapped) in blue, and H041 (Accession:WHPH00000000) in red. Differences between the FA1090 Reference Genome and FA1090 WT primarily consisted of phage-associated genes and hypothetical proteins (~90%). The remainder were derived from a combination of manually curated gene calls of the FA1090 reference genome, automated gene calls for FA1090 WT, and default ortholog-determination cutoffs by PanOCT. Note that paralogs were not excluded for this particular analysis.
