# Supplementary material for: Dual species transcriptomics reveals conserved metabolic and immunologic processes in interactions between human neutrophils and Neisseria gonorrhoeae
Source: PLoS Pathog. 2024 Jul 8;20(7):e1012369. doi: 10.1371/journal.ppat.1012369 (PMC11257400; doi:10.1371/journal.ppat.1012369)
Supplement: S5 Fig — (PDF) [file ppat.1012369.s006.pdf]

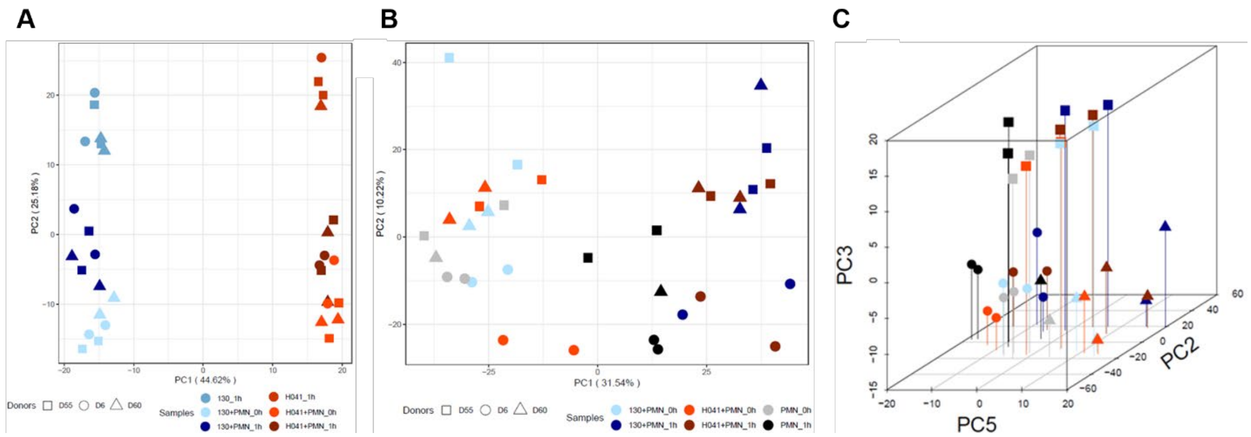

S5 Fig. **Principal Component Analyses (PCA) of Gc and PMN transcriptomes including 0-hour samples.** A) PCA of Gc transcriptomes based on 1582 core genes. PC1 separates the 130 and H041 strains, and PC2 separates the different infection conditions and timepoints. B) PCA of PMN transcriptomes. PC1 separates the two timepoints (0h vs 1h), and PC2 separates the different PMN donors. C) 3-dimensional PCA based on the top 1% of genes contributing to PMN principal components (PC2, PC3 & PC5, genes listed in **S2 Dataset**).
