# Supplementary material for: Dual species transcriptomics reveals conserved metabolic and immunologic processes in interactions between human neutrophils and Neisseria gonorrhoeae
Source: PLoS Pathog. 2024 Jul 8;20(7):e1012369. doi: 10.1371/journal.ppat.1012369 (PMC11257400; doi:10.1371/journal.ppat.1012369)
Supplement: S6 Fig — (PDF) [file ppat.1012369.s007.pdf]

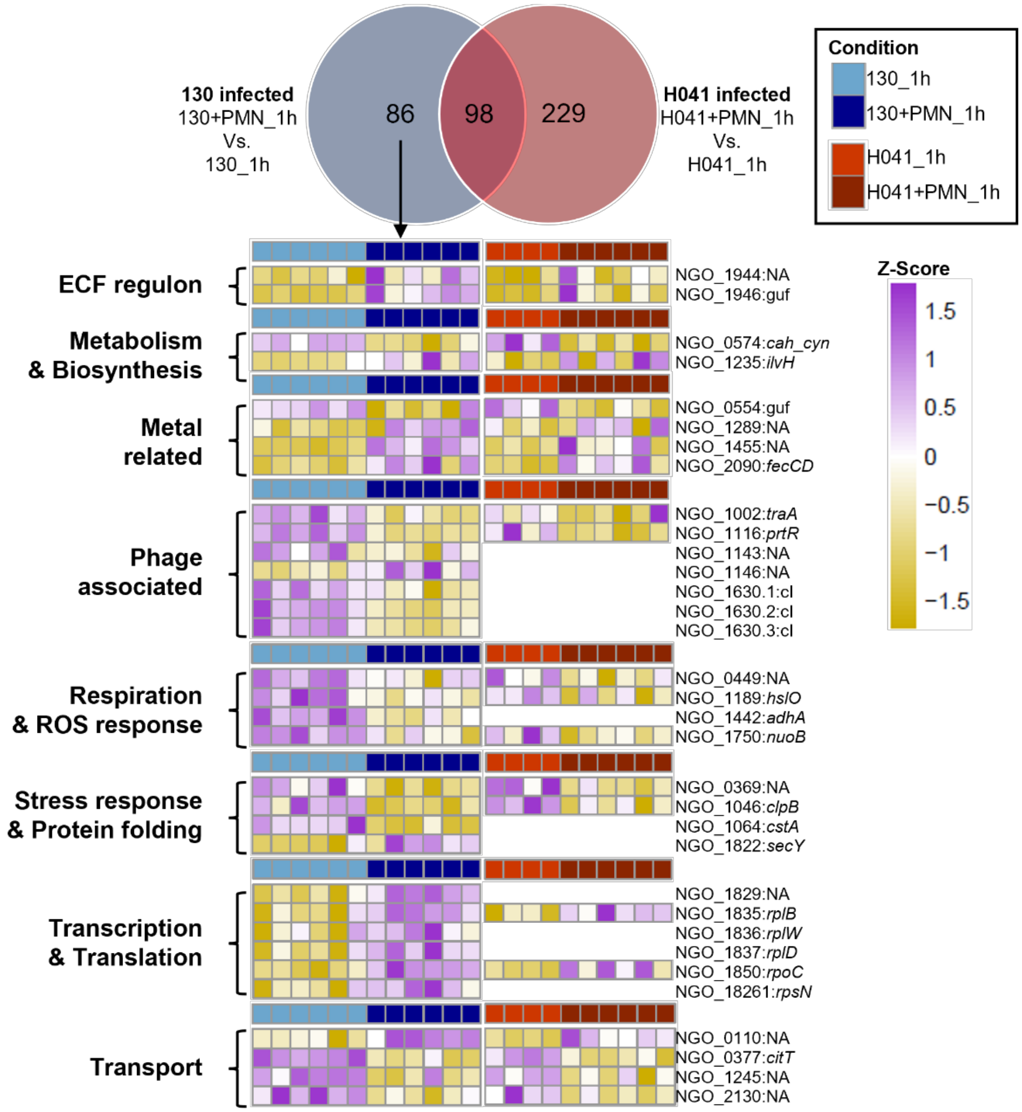

S6 Fig. Heatmaps of Gc regulons enriched by FA1090 Opaless 130 specific DE genes. Z-scored expression levels of FA1090 Opaless 130 genes and FA1090 WT locus tags (F9Z35, Genbank WHPG00000000) are shown (arrow) along with their H041 orthologs, demonstrating that genes that are conserved in both strains of Gc generally follow the same trend in expression.
