# Supplementary material for: Dual species transcriptomics reveals conserved metabolic and immunologic processes in interactions between human neutrophils and Neisseria gonorrhoeae
Source: PLoS Pathog. 2024 Jul 8;20(7):e1012369. doi: 10.1371/journal.ppat.1012369 (PMC11257400; doi:10.1371/journal.ppat.1012369)
Supplement: S7 Fig — (PDF) [file ppat.1012369.s008.pdf]

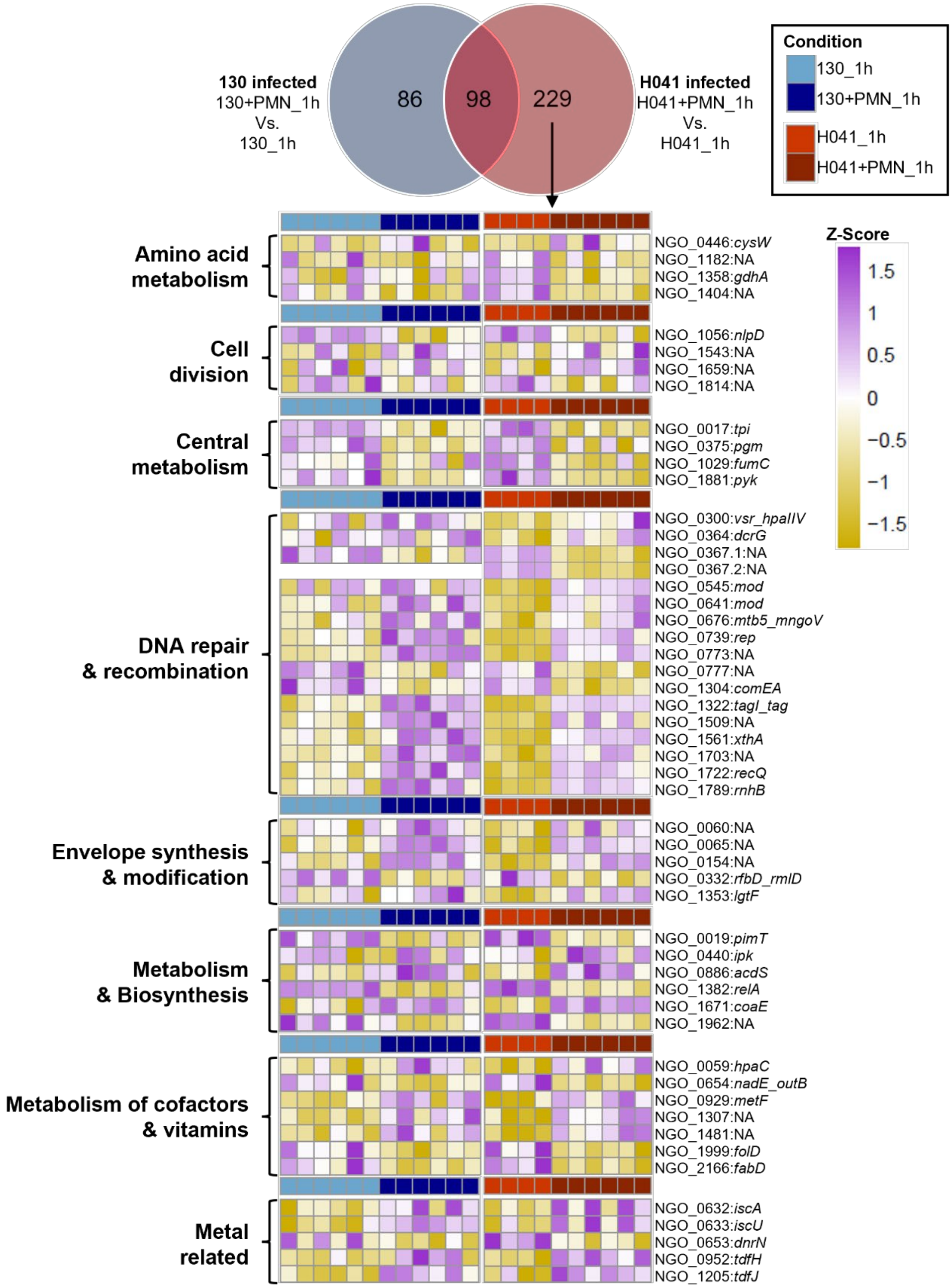
130 infected
130+PMN_1h
Vs.
130_1h
86
98
229
H041 infected
H041+PMN_1h
Vs.
H041_1h
Condition
130_1h
130+PMN_1h
H041_1h
H041+PMN_1h
Z-Score
1.5
1
0.5
0
−0.5
−1
−1.5
Amino acid metabolism
NGO_0446:cysW
NGO_1182:NA
NGO_1358:gdhA
NGO_1404:NA
Cell division
NGO_1056:nlpD
NGO_1543:NA
NGO_1659:NA
NGO_1814:NA
Central metabolism
NGO_0017:tpi
NGO_0375:pgm
NGO_1029:fumC
NGO_1881:pyk
DNA repair & recombination
NGO_0300:vsr_hpaIIV
NGO_0364:dcrG
NGO_0367.1:NA
NGO_0367.2:NA
NGO_0545:mod
NGO_0641:mod
NGO_0676:mtb5_mngoV
NGO_0739:rep
NGO_0773:NA
NGO_0777:NA
NGO_1304:comEA
NGO_1322:tagI_tag
NGO_1509:NA
NGO_1561:xthA
NGO_1703:NA
NGO_1722:recQ
NGO_1789:rnhB
Envelope synthesis & modification
NGO_0060:NA
NGO_0065:NA
NGO_0154:NA
NGO_0332:rfbD_rmlD
NGO_1353:lgtF
Metabolism & Biosynthesis
NGO_0019:pimT
NGO_0440:ipk
NGO_0886:acdS
NGO_1382:relA
NGO_1671:coaE
NGO_1962:NA
Metabolism of cofactors & vitamins
NGO_0059:hpaC
NGO_0654:nadE_outB
NGO_0929:metF
NGO_1307:NA
NGO_1481:NA
NGO_1999:folD
NGO_2166:fabD
Metal related
NGO_0632:iscA
NGO_0633:iscU
NGO_0653:dnrN
NGO_0952:tdfH
NGO_1205:tdfJ

263

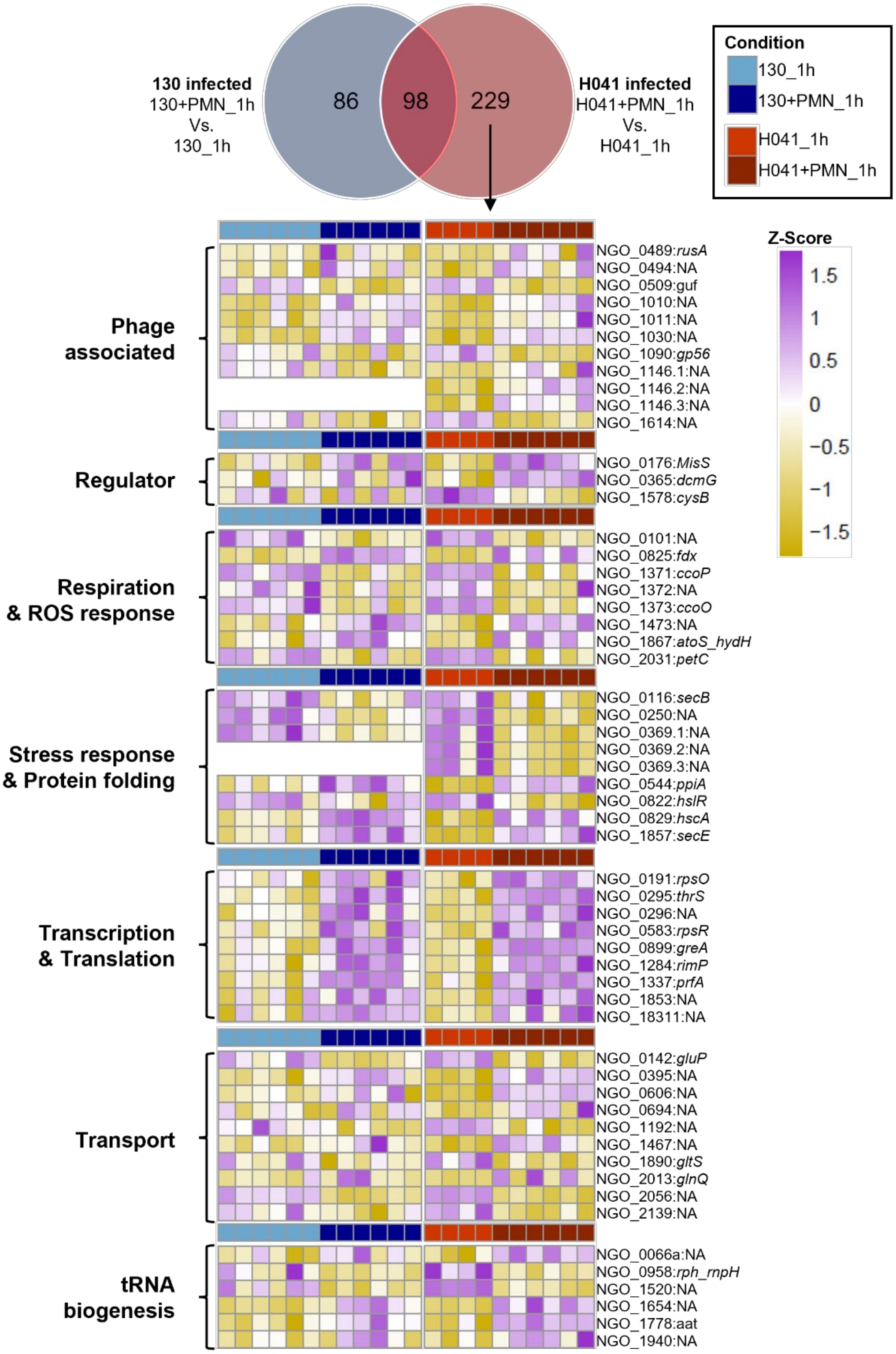

S7 Fig. Heatmaps of Gc regulons enriched by H041 specific DE genes. Z-scored expression levels of H041 genes and their locus tags (F9Z36, Genbank WHPH00000000) are shown (arrow)

along with their FA1090 orthologs, demonstrating that genes that are conserved in both strains of Gc generally follow the same trend in expression.
