# Supplementary material for: Dual species transcriptomics reveals conserved metabolic and immunologic processes in interactions between human neutrophils and Neisseria gonorrhoeae
Source: PLoS Pathog. 2024 Jul 8;20(7):e1012369. doi: 10.1371/journal.ppat.1012369 (PMC11257400; doi:10.1371/journal.ppat.1012369)
Supplement: S8 Fig — (PDF) [file ppat.1012369.s009.pdf]

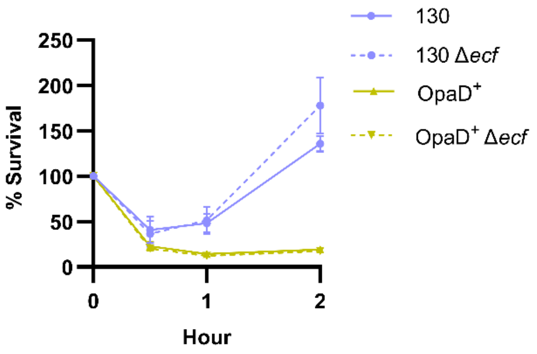

S8 Fig. Ecf does not contribute to survival of Gc from PMNs. Gc strains FA1090 Opaless 130, a constitutive OpaD expressing strain, and isogenic Δ*ecf* mutants were exposed to adherent, IL-8-treated primary human PMNs. Percent Gc survival was calculated by enumerating colony-forming units (CFU) from PMN lysates at 30, 60, and 120 min and reported as the percent of CFU for that strain at 0 min. Significance was determined by one-way ANOVA with Holm–Sidak correction for multiple comparisons. No significant difference was observed between Δ*ecf* mutants and the Opaless and $OpaD^+$ parent. n = 2 independent experiments.
