# Supplementary material for: Dual species transcriptomics reveals conserved metabolic and immunologic processes in interactions between human neutrophils and Neisseria gonorrhoeae
Source: PLoS Pathog. 2024 Jul 8;20(7):e1012369. doi: 10.1371/journal.ppat.1012369 (PMC11257400; doi:10.1371/journal.ppat.1012369)
Supplement: S9 Fig — (PDF) [file ppat.1012369.s010.pdf]

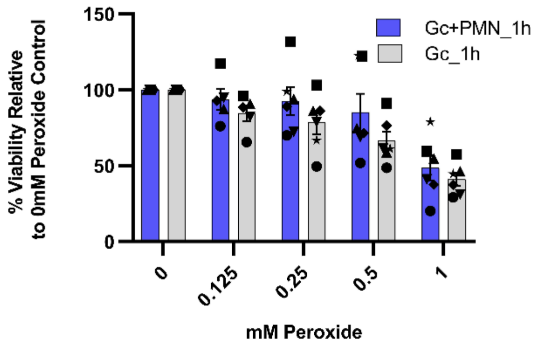


**<u>S9 Fig.</u> Gc resistance to hydrogen peroxide is enhanced by exposure to PMNs.** Gc strain FA1090 Opaless 130 was inoculated onto IL-8-treated, adherent human PMNs or a media alone control, and incubated for 1 h. PMNs were treated with 1% saponin to liberate intracellular bacteria for 10 min before harvesting. Bacteria were washed and resuspended in GCBL at $10^7$ CFU/ml. Gc recovered from the PMNs (Gc+PMN_1h) or from growth in media alone (Gc_1h) were then exposed to 1, 0.5, 0.25, 0.125 or 0 mM $H_2O_2$ for 15 min, before quenching with catalase and plating at limiting dilution for CFUs. Shapes are data points from six donors' PMNs. Bars represent the mean ± SEM. *n* = 5-6 independent experiments. Linear mixed effects modelling fit by Restricted Maximum Likelihood (REML) was used to assess the significance of PMN pre-treatment and $H_2O_2$ concentration in explaining variations in percent viability, with PMN pre-treatment and $H_2O_2$ Concentration as fixed effects and donor as a random effect: Viability ~ Pre-Treatment + (1 | Donor) + $H_2O_2$ Concentration. Intercept (92.893*** ± 7.055), PMN Treatment (12.610*** ± 3.284), $H_2O_2$ Concentration (-53.225*** ± 4.940). The increase in Gc survival from $H_2O_2$ with PMN pretreatment was statically significant by REML (***p<0.001, not shown on graph).
