# Supplementary material for: Dual species transcriptomics reveals conserved metabolic and immunologic processes in interactions between human neutrophils and Neisseria gonorrhoeae
Source: PLoS Pathog. 2024 Jul 8;20(7):e1012369. doi: 10.1371/journal.ppat.1012369 (PMC11257400; doi:10.1371/journal.ppat.1012369)
Supplement: S10 Fig — (PDF) [file ppat.1012369.s011.pdf]

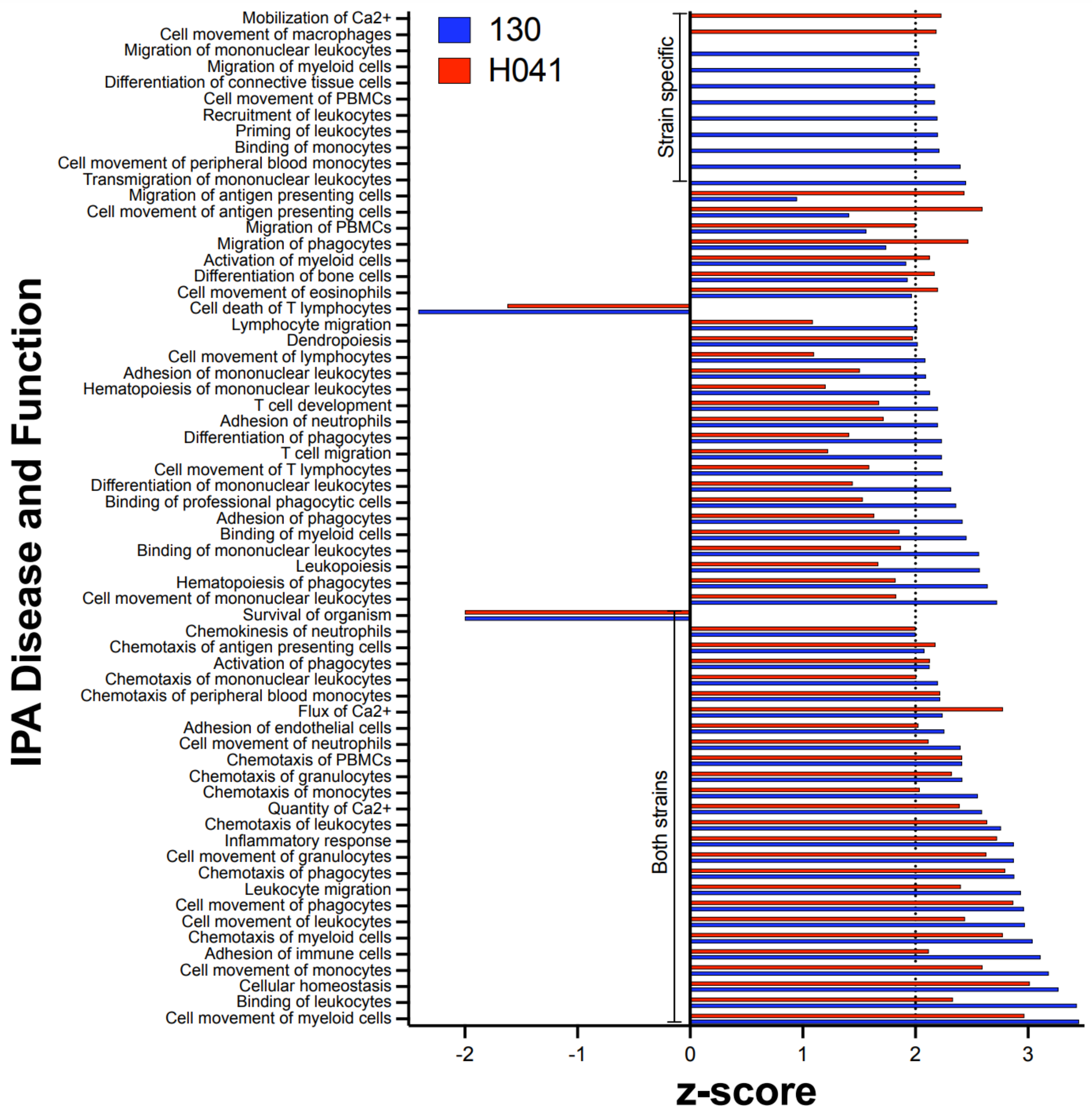

<u>S10 Fig.</u> Ingenuity Pathway Analysis (IPA)-enriched Disease and Functions. Utilizing immune cells specific parameters, resultant functions of PMN DE genes at 1h exposure to GC vs 1h unexposed for FA1090 Opaless 130 (blue), H041 (red) were plotted. The IPA determined Z-score is considered significant at 2 (dotted line). Pathways are grouped based on those exclusive to one strain, significant in one strain and present but not reaching significance in the other, and significantly regulated in both strains.
