# Supplementary material for: Dual species transcriptomics reveals conserved metabolic and immunologic processes in interactions between human neutrophils and Neisseria gonorrhoeae
Source: PLoS Pathog. 2024 Jul 8;20(7):e1012369. doi: 10.1371/journal.ppat.1012369 (PMC11257400; doi:10.1371/journal.ppat.1012369)
Supplement: S11 Fig — (PDF) [file ppat.1012369.s012.pdf]

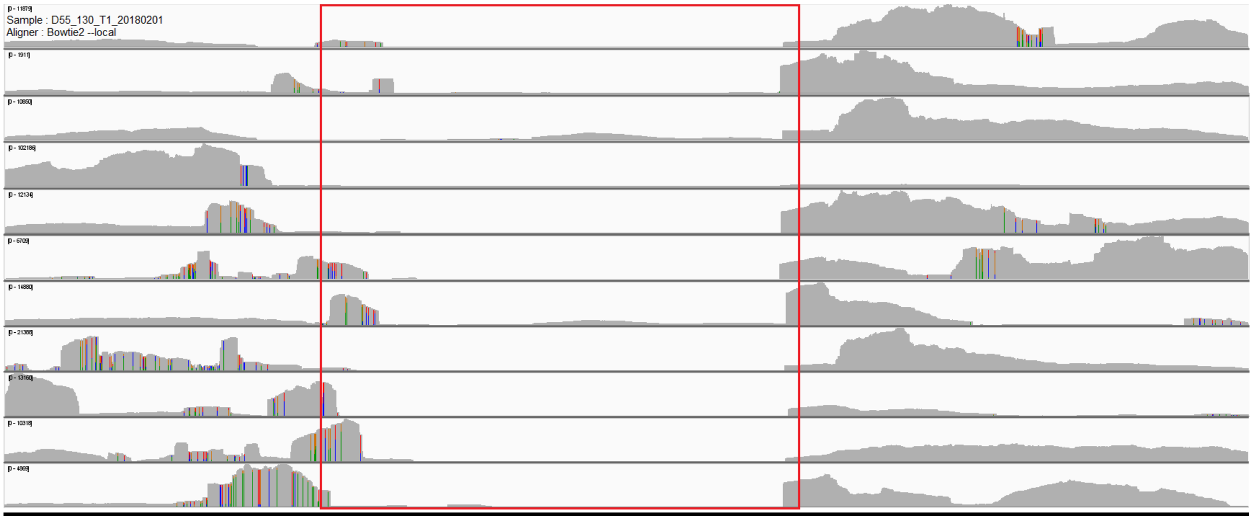

<u>S11 Fig</u>. RNA-Seq coverage of Opa gene deletions for FA1090 Opaless 130 strain seen in Integrated Genomics Viewer (IGV) on an FA1090 WT background. All Opa gene deletions had little to no coverage in the deleted regions, as expected. The red box highlights the general deletion region of all Opa genes. Some coverage was observed for OpaF (3rd row) and OpaH (7th row), however these did not span the entire length of the protein and had low mapping quality.
